# The dual Ras-association domains of *Drosophila* Canoe have differential roles in linking cell junctions to the cytoskeleton during morphogenesis

**DOI:** 10.1242/jcs.263546

**Published:** 2024-12-11

**Authors:** Emily D. McParland, Noah J. Gurley, Leah R. Wolfsberg, T. Amber Butcher, Abhi Bhattarai, Corbin C. Jensen, Ruth I. Johnson, Kevin C. Slep, Mark Peifer

**Affiliations:** ^1^ Department of Biology, University of North Carolina at Chapel Hill, CB#3280, Chapel Hill, NC 27599-3280, USA; ^2^ Biology Department, Wesleyan University, Middletown, CT 06459, USA; ^3^ Lineberger Comprehensive Cancer Center, University of North Carolina at Chapel Hill, Chapel Hill, NC 27599, USA

**Keywords:** Adherens junction, Canoe, Afadin, Rap1, GTPases, Morphogenesis

## Abstract

During development cells must change shape and move without disrupting dynamic tissue architecture. This requires robust linkage of cell–cell adherens junctions to the force-generating actomyosin cytoskeleton. *Drosophila* Canoe and mammalian afadin play key roles in the regulation of such linkages. One central task for the field is defining mechanisms by which upstream inputs from Ras-family GTPases regulate Canoe and afadin. These proteins are unusual in sharing two tandem Ras-association (RA) domains – RA1 and RA2 – which when deleted virtually eliminate Canoe function. Work *in vitro* has suggested that RA1 and RA2 differ in GTPase affinity, but their individual functions *in vivo* remain unknown. Combining bioinformatic and biochemical approaches, we find that both RA1 and RA2 bind to active Rap1 with similar affinities, and that their conserved N-terminal extensions enhance binding. We created *Drosophila canoe* mutants to test RA1 and RA2 function *in vivo*. Despite their similar affinities for Rap1, RA1 and RA2 play strikingly different roles. Deleting RA1 virtually eliminates Canoe function, whereas mutants lacking RA2 are viable and fertile but have defects in junctional reinforcement in embryos and during pupal eye development. These data significantly expand our understanding of the regulation of adherens junction–cytoskeletal linkages.

## Teaser

**Summary:** Rap1 regulation of *Drosophila* Canoe and its role in linking cell junctions to the cytoskeleton during morphogenesis involves two Ras-association (RA) domains that have very different functions

## INTRODUCTION

Small GTPases regulate diverse processes ranging from cell signaling to protein trafficking and cytoskeletal regulation (reviewed in Cherfils and Zeghouf, 2013). One key challenge for the field is uncovering the mechanisms by which these GTPases regulate their protein effectors. Members of the Ras family of GTPases, including K-Ras, N-Ras, H-Ras and Rap1, often bind regulators and effectors via ubiquitin-fold Ras-association (RalGDS/AF6; referred to hereafter as RA) domains (Smith, 2023; Wohlgemuth et al., 2005). Defining the mechanisms by which these interactions regulate effector function are important goals for our field.

**Fig. 1.**
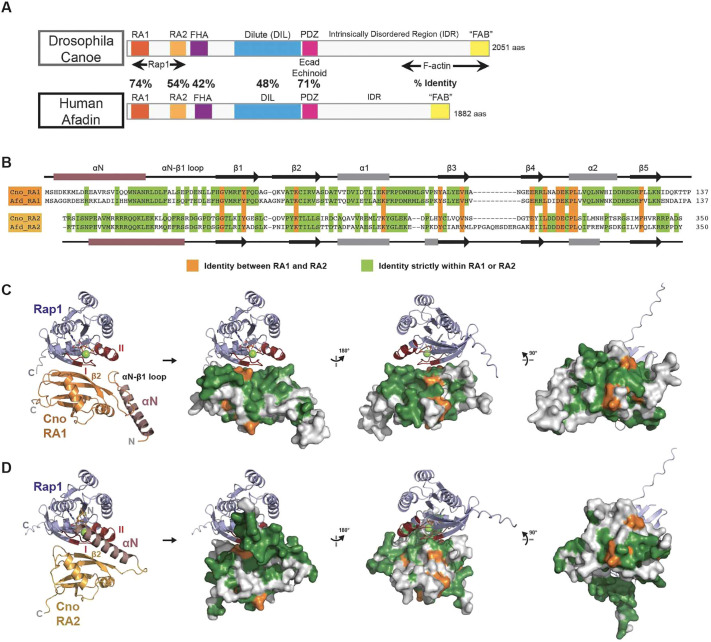
**The Cno RA domains are predicted to bind Rap1 using a conserved surface but have unique conserved surfaces outside that binding site.** (A) *Drosophila* Cno and human afadin. Regions involved in interactions with Rap1, F-actin, and E-cadherin (Ecad) and Echinoid are marked, along with amino acid sequence identity for selected domains (aas, amino acids). (B) Sequence alignment and secondary structure of *Drosophila* Cno and mouse afadin (Afd) RA1 and RA2. αN helices (αN) are colored in purple. Green, residues identical strictly within either RA1 or RA2; orange, residues identical between RA1 and RA2. (C,D) AlphaFold2 models of Cno RA1–Rap1 (C) and Cno RA2–Rap1 (D) complexes. Left: cartoon format. Right three panels: RA domain in surface representation, with identity mapped as in B, rotated as shown. The αN helix of each RA domain is colored in purple. Rap1 switch I and II regions are shown in dark red. GMPPNP and Mg^2+^ are modeled based on aligning predictions with the afadin RA1–H-Ras structure (PDB ID: 6AMB; Fig. S1C).

We focus on mechanisms regulating cell shape change and movement during morphogenesis and the protein network mediating this by linking cell–cell adherens junctions (AJs) to actin and myosin (Perez-Vale and Peifer, 2020). *Drosophila* Canoe (Cno) and its mammalian homolog afadin (referred to here collectively as Cno/afadin) are adapter proteins that are key components of this network. They regulate diverse events ranging from initial apical AJ positioning (Choi et al., 2013) to cell shape changes of gastrulation and convergent elongation (Sawyer et al., 2011, 2009), as well as collective cell movement during dorsal closure and head involution (Boettner et al., 2003; Choi et al., 2011). Both are complex multidomain proteins sharing five folded protein domains and a long C-terminal intrinsically disordered region (IDR) (Fig. 1A; Gurley et al., 2023). Most N-terminal of these are two RA domains (RA1 and RA2), consistent with idea that Cno/afadin are Ras-family GTPase effectors.

Rap1 acts through multiple effectors to regulate cell–cell and cell–matrix adhesion (Boettner and Van Aelst, 2009). Fly and human Rap1 proteins share ∼55% sequence identity with canonical Ras proteins, and in their activated state bind RA domains of multiple effectors *in vitro* (Wohlgemuth et al., 2005). These range from integrin adhesion regulators like RIAM (also known as APBB1IP) to cytoskeletal regulators like Tiam proteins and AJ–cytoskeletal linkers like Cno/afadin.

Afadin was identified through biochemical searches for canonical Ras binding partners (Kuriyama et al., 1996; Van Aelst et al., 1994), and it binds *in vitro* to multiple small GTPases including Rap1 (Boettner et al., 2000), M-Ras (Quilliam et al., 1999), Rit (RIT1) and Rin (RIT2) (Shao et al., 1999). However, functional analysis in *Drosophila* has revealed that Rap1 is the key Cno regulator in events ranging from establishment of apical–basal polarity (Choi et al., 2013) to mesoderm invagination (Sawyer et al., 2009; Spahn et al., 2012) and dorsal closure (Boettner et al., 2003). Consistent with this, deleting both RA domains compromises all Cno functions (Perez-Vale et al., 2021). Rap1 also acts through mammalian afadin in multiple contexts from kidney tubule lumen formation (Hiremath et al., 2023) to angiogenesis and barrier recovery after vascular injury (Birukova et al., 2013; Tawa et al., 2010), and deleting both RA domains reduces afadin function (Choi et al., 2016). Although other members of the Ras family can regulate Cno/afadin in some contexts (Iwasawa et al., 2012; Gaengel and Mlodzik, 2003; Matsuo et al., 1997), the bulk of the evidence supports a key role for Rap1 in regulating Cno function and suggests that Rap1 acts, at least in part, via the RA domains of Cno (Perez-Vale et al., 2021). However, the mechanisms by which this occurs remain unknown.

Whereas most Ras-family GTPase effectors and regulators have only a single RA domain, Cno/afadin has two RA domains positioned in tandem (Fig. 1A). Given the essential role of the RA region in Cno function, it is crucial to define roles of each of the two RA domains in Rap1 binding and Cno function, defining whether they serve similar or different functions. RA1 is very well conserved: Cno and afadin RA1 domains share 74% amino acid identity (Fig. 1A,B; Gurley et al., 2023). Intriguingly, whereas most RA domains have a ββαββαβ ubiquitin fold (Mott and Owen, 2015), the RA1 domain of afadin, like the RASSF5 RA domain, has an additional N-terminal α-helix (Smith et al., 2017). Sequence conservation suggests that this feature is shared by Cno RA1. RA2 is less well conserved (54% amino acid identity *s*hared by Cno and afadin; Fig. 1A,B). Alphafold (Jumper et al., 2021; Varadi et al., 2022) predicts that the afadin and Canoe RA2 domains also have an additional N-terminal α-helix (Fig. 1C). However, although RA1 and RA2 are predicted to fold into similar structures, they are very divergent in sequence, with less than 30% sequence identity between the Cno RA1 and RA2 domains (Fig. 1B).

Consistent with this, previously reported data suggest that the Cno/afadin RA1 and RA2 domains have diverged in their interactions with Ras GTPases. Afadin RA1 has been found to have a high affinity for Rap1 (*K*_d_=0.24 µM), a moderate affinity for H-Ras, M-Ras or Rap2 (*K*_d_=2–3 µM), and low affinity for R-Ras and TC21 (also known as RRAS2; *K*_d_=16 µM; Linnemann et al., 1999; Wohlgemuth et al., 2005). In contrast, the same researchers found that afadin RA2 has a very low affinity for H-Ras (*K*_d_=30 µM) and no detectable binding to Rap1 (Wohlgemuth et al., 2005). These measurements of afadin RA2 interactions used a construct lacking the N-terminal α-helix, whereas the parallel assays of RA1 included the N-terminal α-helix (Wohlgemuth et al., 2005). However, recent work suggests that this difference between the constructs used might contribute to the observed difference in Ras GTPase interactions. Ikura's lab have examined afadin RA1 with or without its N-terminal α-helix, finding that deletion of the helix reduces affinity for Ras proteins 4-fold, from a *K*_d_ of 4.1 µM to a *K*_d_ of 17.8 µM (Smith et al., 2017). Analyses of the Cno RA domains are less comprehensive. The results of yeast-two hybrid assays suggest that Rap1 binds both Cno RA1 and RA2, and imply a stronger interaction with RA1; however, affinities were not measured (Boettner et al., 2003). These data suggest hypotheses for RA domain function based on differences in Rap1 affinity.

Phospholipase C epsilon (PLCε, also known as PLCE1) also has two RA domains (Dusaban and Brown, 2015), and the individual functions of each have been explored in detail. PLCε integrates signaling from G-protein-coupled receptors to downstream kinases by generating lipid second messengers and acting as a guanine-nucleotide-exchange factor (GEF) for Rap1. The two PLCε RA domains have a C-terminal location. Like RA1 of Cno/afadin, the PLCε RA2 domain binds small GTPases, having ∼8-fold higher affinity for Ras proteins than for Rap1 (Bunney et al., 2006). RA2 mediates both PLCε plasma membrane recruitment and activation by Ras proteins and Rap1 (Bunney et al., 2006; Kelley et al., 2001; Song et al., 2002). In contrast, the PLCε RA1 domain does not bind these GTPases (Bunney et al., 2006). Instead, structural studies have revealed that RA1 mediates intradomain interactions promoting PLCε stability and basal activity (Rugema et al., 2020).

These data suggest multiple mechanistic hypotheses for how Rap1 regulates Canoe via its dual RA domains. For example, these domains might be functionally redundant with similar affinities for Rap1, they might have different affinities for Rap1, or one might serve a more structural function. We tested these hypotheses, combining biochemical assessment of Rap1 binding with genetic and cell biological analyses of RA domain functions *in vivo*.

## RESULTS

### Conserved regions N-terminal to each RA domain are predicted to potentiate Rap1 binding

*Drosophila* Cno and mammalian afadin share substantial sequence identity between their RA domains (74% for RA1, 54% for RA2; Fig. 1A,B, residues in green). However, RA1 and RA2 are much less similar to one another, with only 14% amino acid sequence identity over the canonical RA domain (Fig. 1B, residues in orange). Some hypotheses for the mechanistic function of the two RA domains rest on data suggesting a high affinity between activated Rap1 and RA1, and weaker or no binding to RA2. We thus used AlphaFold2 to model Cno RA1–Rap1 and Cno RA2–Rap1 complexes, to reveal potential structural attributes that might underlie affinity differences and highlight potential functional regions that RA1 and RA2 share. As the region N terminal to each RA domain is conserved, and because the predicted RA1 N-terminal α-helix increases affinity for activated Ras (Smith et al., 2017), we included these regions in the AlphaFold2 inputs. AlphaFold2 generated structural models with high confidence [Fig. S1A,B; see plots of predicted local distance difference test (pLDDT) values], with Rap1 engaging both RA1 and RA2 using switch regions I and II in their active GTP-bound state, and the C-terminal β-strand of switch I engaging the β2 strand of each RA domain in an anti-parallel interaction. The predicted interactions align well with the crystal structure of mouse afadin RA1 bound to activated H-Ras [root mean square deviation (RMSD)=0.70 Å over 147 Cα atoms; Fig. S1C; Smith et al., 2017]. The Cno RA1–Rap1 and Cno RA2–Rap1 complexes also align well with each other (RMSD=1.08 Å over 204 Cα atoms; Fig. S1D).

Interestingly, each model suggested that a predicted α-helix located N-terminally of the RA domain (referred to hereafter as the αN helix) supports Rap1 interaction in different ways. The αN helix preceding RA1 docks alongside the core RA domain, stabilizing the conserved αN–β1 loop that is modeled contacting Rap1 switch II (Fig. 1C). Although this N-terminal region is not fully present or ordered in the afadin RA1 construct crystallized with H-Ras (Fig. S1C), the model aligns with biochemical data revealing that the αN helix potentiates Ras protein binding (Smith et al., 2017). The Cno RA2–Rap1 model also predicts an αN helix (Fig. 1D). Its conformation is distinct from that in RA1. Consistent with this, there is no sequence identity between the αN helices of RA1 and RA2 (Fig. 1B–D). In the Cno RA2–Rap1 model, the αN helix is not docked alongside the RA domain, but instead contacts the Rap1 switch I region and might engage the GTP ribose (Fig. 1D). Mapping identity highlights distinct conserved RA1 versus RA2 residues at the Rap1 binding site that might lead to differences in affinity (Fig. 1C,D, right three panels). RA domain-specific conserved sequences also map to regions outside the Rap1 binding site, including the relative back face of each RA domain. These conserved regions of RA1 and RA2 differ, suggesting that each RA domain might, by itself or when bound to a GTPase, engage distinct targets, providing another possible mechanism by which these domains could differentially regulate Cno function.

### The Cno RA domains both bind activated Rap1 with similar affinities

**Fig. 2.**
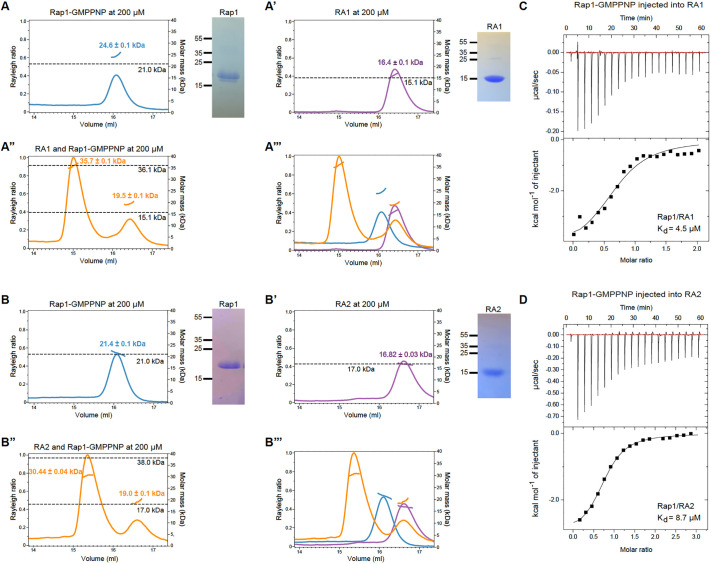
**Activated Rap1 forms stable complexes with both RA1 and RA2.** (A–B‴) SEC-MALS of *Drosophila* Rap1 and Cno RA domains alone and in complex. Formula molecular masses for GMPPNP-bound Rap1G12V, RA1 and RA2, and for 1:1 stoichiometric Rap1–RA domain complexes, are indicated in the respective panels by dashed horizontal lines and were confirmed with SDS-PAGE (right-hand images are Coomassie Brilliant Blue-stained gels, molecular mass marked in kDa). Experimental masses were determined by SEC-MALS. SEC-MALS was performed in duplicate. A representative run is shown, with experimental mass data overlaid on the chromatograms. (A) Rap1-GMPPNP-Mg: formula mass=21.0 kDa, experimental mass=24.6±0.1 kDa. (A′) RA1: formula mass=15.1 kDa (His-tag removed), experimental mass=16.4±0.1 kDa. (A″) The complex: formula mass=36.1 kDa, experimental mass=35.7±0.1 kDa (left peak). Unbound RA1 (right peak): experimental mass=19.5±0.1 kDa. (A‴) Super-positioning of runs in A–A″. (B) Rap1-GMPPNP-Mg: formula mass=21.0 kDa, experimental mass=21.4±0.1 kDa. (B′) RA2 with His-tag: formula mass=17.0 kDa, experimental mass=16.82±0.03 kDa. (B″) The complex: formula mass=38.0 kDa, experimental mass=30.44±0.04 kDa (left peak). Unbound RA2 (right peak): experimental mass=19.0±0.1 kDa. (B‴) Super-positioning of runs in B–B″. Errors are s.e.m. (C,D) ITC analyses of interactions between Rap1-GMPPNP and RA1 (C) or RA2 (D). Data from representative experiments are shown. Rap1–RA1 ITC and Rap1–RA2 ITC were performed in duplicate and triplicate, respectively. Detailed results of each experiment are in the Materials and Methods.

AlphaFold2 predicted robust models of Cno RA–Rap1 complexes for both RA1 and RA2. We next tested the hypothesis that the distinct conservation and unique N-terminal segments affect Rap1 binding affinity, potentially underlying unique roles in Cno function. To test potential binding differences of Cno RA1 or RA2 to activated Rap1, we employed two assays: size exclusion chromatography with multiangle light scattering (SEC-MALS) and isothermal titration calorimetry (ITC). We purified the constitutively active Rap1G12V mutant (which hydrolyzes GTP slowly) and further stabilized the active state by exchanging in the non-hydrolyzable nucleotide GMPPNP. We first used SEC-MALS to analyze Rap1-GMPPNP and RA1 alone and in complex. Each individual protein eluted as a single peak with an experimental mass near its formula mass (Fig. 2A,A′). When we incubated stoichiometric amounts of Cno RA1 and Rap1-GMPPNP, a distinct peak eluted earlier than the individual proteins, and this peak had an experimental mass of 35.7 kDa, which is close to the 1:1 complex formula mass of 36.1 kDa (Fig. 2A″,A‴). Some excess RA1 eluted at 16.4 ml, likely due to errors in measuring stoichiometry (Fig. 2A″). The robust elution shift of the complex, the profile of the peak, and the match between experimental and formula masses indicates that interaction between RA1 and activated Rap1 is robust over the course of gel filtration (flow rate: 0.5 ml/min).

We next performed similar experiments using RA2 and Rap1-GMPPNP. Again, individual proteins eluted as single peaks with experimental masses near the formula masses (Fig. 2B,B′). When we incubated stoichiometric amounts of RA2 and Rap1-GMPPNP, an earlier-shifted peak again appeared with an experimental mass of 30.4 kDa, which is greater than the mass of each individual component but somewhat smaller than the formula mass of the complex (38.0 kDa; Fig. 2B″,B‴). Some excess RA2 domain eluted at the 16.6 ml point, again likely due to errors in measuring stoichiometry (Fig. 2B″). Although the RA2–Rap1 complex had a dramatic peak shift relative to the individual proteins, the peak did not shift as much as that of the RA1–Rap1 complex, nor did the experimental mass fully match the formula mass of the complex. This suggests some dissociation of the complex during the run, yielding mixed species and a lower apparent mass.

We next analyzed Rap1-GMPPNP binding affinity to each Cno RA domain using ITC. Rap1-GMPPNP bound Cno RA1 with a *K*_d_ of 4.5 µM (Fig. 2C) and bound Cno RA2 with a *K*_d_ of 8.7 µM (Fig. 2D). The Rap1–RA1 affinity is similar to the reported affinities between afadin RA1 and Ras and Rap GTPases; for example, Smith et al. (2017) have reported a *K*_d_ of 4.1 µM for Ras, and Wohlgemuth et al. (2005) have reported *K*_d_ values of 0.24 µM for Rap1 and 2–3 µM for H-Ras, M-Ras and Rap2. Although the Rap1–RA2 affinity is ∼2-fold weaker than that of Rap1–RA1 in our hands, it is still robust and greater than previously reported binding affinities of afadin RA2 (Wohlgemuth et al., 2005). Our higher Rap1–RA2 affinity is likely due to our use of an RA2 construct including the αN helix. Collectively, our SEC-MALS and ITC studies demonstrate that Cno RA1 and RA2 both bind activated Rap1 with micromolar affinity, and that RA1 has somewhat higher affinity than RA2. This aligns with predictions from our structural models in which common and distinct features of each RA domain are predicted to engage Rap1 and rules out hypotheses in which only one of the RA domains binds Rap1.

### Creating mutants to test functions of the two Cno RA domains

**Fig. 3.**
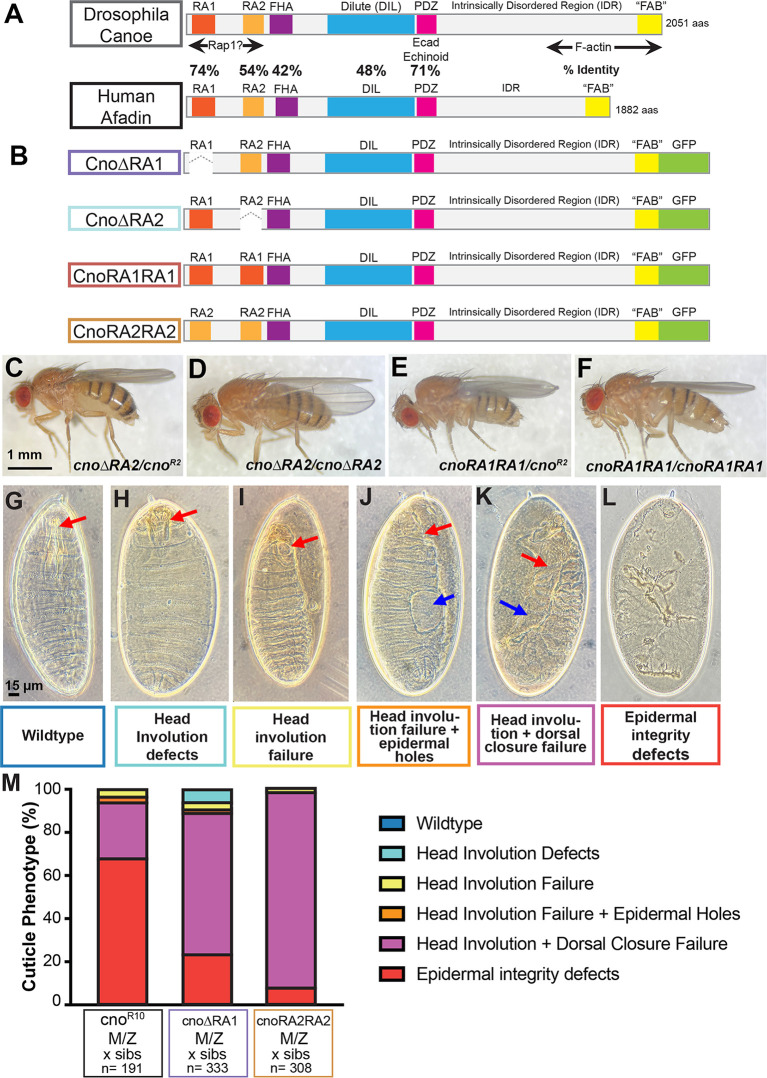
**Deleting RA1 severely reduces Cno function in morphogenesis, RA2 is dispensable and the RA1 domain cannot be replaced by RA2.** (A) *Drosophila* Cno and human afadin, labeled as in Fig. 1A. (B) The Cno mutants examined. (C–F) Adult flies of the indicated genotypes. *cno*Δ*RA2* and *cnoRA1RA1* are viable over the null allele *cno^R2^* and are viable when homozygous. Scale bar in C applies to C–F. (G–L) Embryonic cuticles in eggshells at the end of embryonic development, showing illustrative examples of possible defects differing in severity. (G) Wild-type embryo with intact head skeleton (arrow). (H) Defects in head involution disrupt the head skeleton (arrow). (I) Complete head involution failure, leading to an anterior hole (arrow). (J) Head involution failure (red arrow) and holes in dorsal or ventral epidermis (blue arrow). (K) Complete failure of head involution and dorsal closure, leaving cuticles open anterior (red arrow) and dorsally (blue arrow). (L) More severe defects in epidermal integrity. Scale bar in G applies to G–L. (M) Stacked bar graph illustrating defects in different mutants. The indicated mutants were scored according to the phenotype categories in G–L. Most *cno* maternal–zygotic (M/Z) null mutants *(cno^R10^)* combine complete failure of head involution and dorsal closure with additional defects in epidermal integrity. Most *cno*Δ*RA1* maternal–zygotic mutants are slightly less severe, with complete failure of head involution and dorsal closure but without additional epidermal holes. *cnoRA2RA2* maternal–zygotic mutants are similar but on average slightly less severe. Sibs, siblings. Images in G–L are also shown in Fig. 7B–G to illustrate the phenotype categories used in Fig. 7H.

The ability of both RA domains to bind Rap1 ruled out some mechanistic hypotheses. We thus tested the hypothesis that RA1 and RA2 function redundantly. Deleting both RA domains severely reduces all Cno functions and alters Cno localization to nascent AJs and to AJs under elevated tension (Perez-Vale et al., 2021). To define the functions of individual RA domains, we generated mutants lacking one or the other; our deletions included the αN helix predicted to precede each domain (Fig. 3A,B). We replaced the *cno* coding sequence at the endogenous locus with sequences encoding GFP-tagged mutant forms of Cno with specified mutational changes (Perez-Vale et al., 2021). We first designed mutants cleanly deleting either RA1 or RA2 – *cno*Δ*RA1* and *cno*Δ*RA2*, respectively – using AlphaFold structures as a guide. In parallel, we generated mutants in which RA1 was cleanly replaced by a second copy of RA2 (*cnoRA2RA2*) or in which RA2 was replaced by a second copy of RA1 (*cnoRA1RA1*; Fig. 3A,B). These mutants allowed us to test the hypothesis that position rather than sequence underlies function of each RA domain. We verified the mutants by PCR and sequencing of transgenic flies. We next examined protein accumulation using an anti-Cno antibody that recognizes the C-terminal IDR or an anti-GFP antibody to detect the inserted GFP tag. This verified accumulation in embryos of GFP-tagged proteins of the expected sizes at levels roughly similar to those of wild-type Cno (Fig. S2), as observed with previously reported mutant proteins (McParland et al., 2024; Perez-Vale et al., 2021). For CnoΔRA1 and CnoΔRA2 (Fig. S2A–F), which run at the same size as wild-type Cno (the GFP tag and region deleted roughly match in size), we compared levels to those of wild-type GFP-tagged Cno, which accumulates at wild-type levels (Perez-Vale et al., 2021). Both CnoΔRA1 and CnoΔRA2 accumulated at levels slightly lower than that of wild-type GFP-tagged Cno during early embryonic development and at levels slightly higher than wild-type GFP-tagged Cno at later stages (Fig. S2E,F). Although the slightly lower levels of both CnoΔRA1 and CnoΔRA2 at 1-4 h of embryonic development could potentially affect function, the functional differences between *cno*Δ*RA1* and *cno*Δ*RA2* reported below suggest that this is not relevant, and the increase to slightly higher than wild-type levels at later stages when many morphogenetic movements are occurring also makes this less likely. CnoRA1RA1 and CnoRA2RA2 have higher molecular masses than wild-type Cno, so we could directly compare their levels to that of wild-type Cno by comparing the two western blot bands in samples from heterozygotes (Fig. S2G–L). Both CnoRA1RA1 and CnoRA2RA2 accumulated at levels very close to that of wild-type Cno (Fig. S2K,L). Finally, we outcrossed our mutants over multiple generations to a wild-type stock, removing other potential mutations on the third chromosome.

### RA1 is essential for viability, whereas RA2 is dispensable

To initially test mutant protein function, we crossed each mutant to one of our canonical protein-null alleles*, cno^R2^* (Sawyer et al., 2009), and assessed adult viability. *cno*Δ*RA* mutants, which lack both RA domains, are lethal before adulthood (Perez-Vale et al., 2021). Strikingly, whereas *cno*Δ*RA1* was lethal over *cno^R2^, cno*Δ*RA2/cno^R2^* mutants were viable to adulthood at Mendelian ratios (Fig. 3C; 36% viable versus 33% predicted; *n*=736). Consistent with this, maternal–zygotic *cno*Δ*RA2* mutant embryos derived from a homozygous stock (Fig. 3D) exhibited only 2% lethality (*n*=911), which was in the wild-type range. Thus, RA1 is crucial for viability whereas RA2 is dispensable, ruling out the hypothesis that they are redundant.

### RA2 cannot replace RA1

Since both RA1 and RA2 bind Rap1, we tested the hypothesis that they are functionally equivalent, but that the N-terminal position of RA1 is crucial. However, replacing RA1 with a second copy of RA2 (*cnoRA2RA2*) did not rescue the lethality resulting from loss of RA1*.* In contrast, *cnoRA1RA1/cno^R2^* mutants were viable to adulthood at Mendelian ratios (Fig. 3E; 36% versus 33% predicted; *n*=166). Homozygous *cnoRA1RA1* adults (Fig. 3F) were viable and fertile, and *cnoRA1RA1* maternal–zygotic mutant embryos had 10% lethality (*n*=955), very slightly elevated from the wild-type range (3–8% lethality). Thus, RA2 cannot replace RA1, whereas putting a second copy of RA1 in the place of RA2 has little or no effect on Cno function.

### RA1 is essential for embryonic morphogenesis

While lethality is one test of function, mutations widely vary in their effects on morphogenesis, with some lethal mutants retaining substantial function and some viable mutants having subtle defects. To fully assess CnoΔRA1 function, we generated maternal–zygotic mutants using the FLP/FRT/ovoD approach (Chou and Perrimon, 1996), creating females with germlines homozygous mutant for *cno*Δ*RA1,* and crossing them to males heterozygous for *cno*Δ*RA1*. *cno*Δ*RA1* maternal–zygotic mutants were fully embryonic lethal, whereas most embryos receiving a wild-type gene were paternally rescued (53% embryonic lethality; *n*=895). As a first assessment of CnoΔRA1 function in morphogenesis, we inspected cuticles secreted by developing larvae; their features allowed us to examine epidermal epithelial integrity and whether key morphogenetic movements like dorsal closure and head involution were completed. In wild-type embryos (Fig. 3G), the epidermis is intact, completion of head involution allows development of an intact head skeleton (Fig. 3G arrow) and dorsal closure seals the cuticle dorsally. In *cno* zygotic null mutants, maternally contributed Cno is sufficient for many morphogenetic events, and most embryos only have defects in head involution (as depicted in Fig. 3H–J). In contrast, *cno* maternal–zygotic null mutants (*cno^R10^*) have fully penetrant defects in both dorsal closure and head involution (as depicted in Fig. 3K; 94% of dead embryos; Fig. 3M), and most also have defects in ventral epidermal integrity (as depicted in Fig. 3L; 68% of dead embryos; Fig. 3M; Gurley et al., 2023). *cno*Δ*RA* maternal–zygotic mutants, lacking both RA domains, have a phenotype similar to, although slightly less severe than, that of the null mutant – dorsal closure and head involution fail, but defects in ventral epidermal integrity are less frequent (Perez-Vale et al., 2021). We found that *cno*Δ*RA1* maternal–zygotic mutants also had severe disruptions of morphogenesis, with penetrant failure of dorsal closure and head involution (89% of dead embryos); 24% also had additional holes in the ventral epidermis (Fig. 3M). Thus, deleting RA1 leads to a very strong loss of Cno function, with some potential residual function remaining.

To test whether replacing RA1 with RA2 restores any function, we assessed CnoRA2RA2. *cnoRA2RA2* maternal–zygotic mutants were fully embryonic lethal, with strong paternal rescue (54% embryonic lethality; *n*=820). Cuticle phenotypes revealed that CnoRA2RA2 also failed to rescue morphogenesis. Like *cno*Δ*RA1* mutants, the *cnoRA2RA2* maternal–zygotic mutants exhibited nearly fully penetrant dorsal closure and head involution failure (95% of dead embryos; Fig. 3M). We observed that 9% also had additional epidermal integrity defects, versus 24% for *cno*Δ*RA1* (Fig. 3M). Thus, replacing RA1 with a second copy of RA2 does not restore Cno function, ruling out the hypothesis that it is RA1 position alone that matters. The slightly lower frequency of epidermal holes suggests that CnoRA2RA2 might provide a small amount more residual function than CnoΔRA1.

### RA1 deletion disrupts Cno function in initial AJ positioning and dramatically reduces Cno recruitment to nascent AJs

**Fig. 4.**
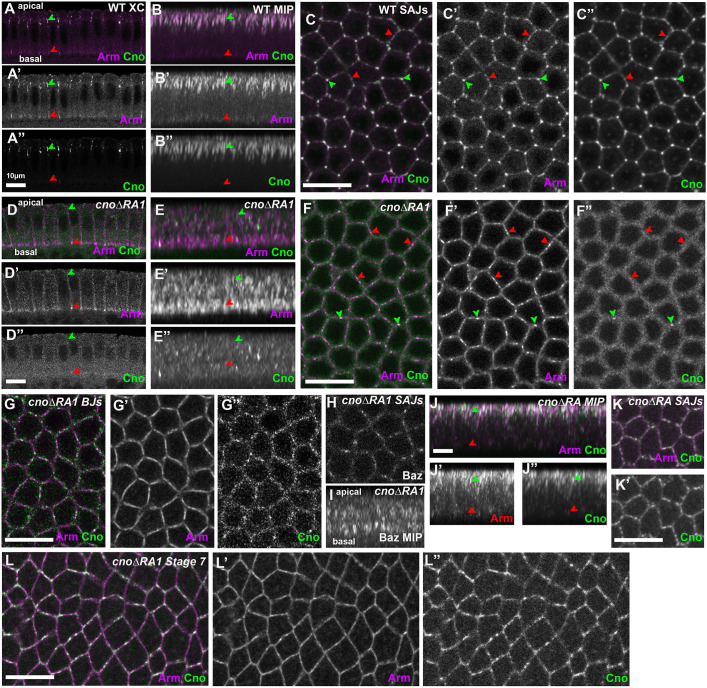
**The RA1 domain is required for Cno localization and function as AJs are positioned during cellularization.** (A–K′) Immunofluorescence images of late cellularization embryos. Genotypes and antigens are indicated. (A–B″) Wild-type (WT) cross-section (XC) (A–A″) and MIP (B–B″). Arm is enriched both in nascent SAJs (green arrowheads) and basal junctions (red arrowheads). Cno localizes to SAJs (green arrowheads) and is largely absent more basally (red arrowheads). (C–C″) Images acquired at the level of SAJs. Wild-type Cno is enriched at TCJs (green arrowheads) relative to bicellular junctions (red arrowheads). (D–E″) *cno*Δ*RA1* cross-section (D–D″) and MIP (E–E′). (F–G″) *cno*Δ*RA1.* Images acquired at the level of SAJs (F–F″) or basal junctions (BJs) (G–G″). Arm enrichment at SAJs is reduced (D–E″ green arrowheads) whereas basal junction enrichment remains (D–E″ red arrowheads, G). CnoΔRA1 protein puncta are found all along the apical–basal axis. Membrane enrichment is reduced (F″,G″). No TCJ enrichment is apparent (arrowheads in F–F″). (H,I) Baz imaged at the level of SAJs (H) and in MIP (I). Normal apical Baz enrichment is lost. (J–K′) *cno*Δ*RA*. Although CnoΔRA protein is less apically enriched, it remains localized to cell membranes (green arrowheads indicate SAJs; red arrowheads more basally localized protein). (L–L″) Immunofluorescence images of a stage 7 *cno*Δ*RA1* embryo. Antigens are indicated. As gastrulation starts, CnoΔRA1 protein returns to AJs. All scale bars: 10 μm. Scale bars in A and D are also accurate for B and E. Scale bar in G is accurate in H and I.

The first role of Cno in morphogenesis occurs as AJs are initially positioned apically during cellularization. As membranes invaginate around nuclei, cadherin–catenin complexes [visualized by labeling Armadillo (Arm), the fly β-catenin] become enriched apically at nascent spot AJs (SAJs; Fig. 4A–C″, see green arrowheads in A and B) along with Bazooka (Baz, the fly Par3; Harris and Peifer, 2004). Lower levels of cadherin–catenin complexes accumulate along the lateral membrane, and they are enriched at basal junctions (Hunter and Wieschaus, 2000) just behind actin at the cellularization front (Fig. 4A,B red arrowheads). This is most apparent in maximum-intensity projections (MIPs) of multiple cross sections (Fig. 4B). Cno localizes to the apico-lateral SAJs (Fig. 4A,B green arrowheads; Choi et al., 2013), accumulating in all SAJs but particularly enriched at tricellular junctions (TCJs; Fig. 4C–C″, red versus green arrowheads; Bonello et al., 2018). In the absence of Cno, both Arm and Baz lose apical enrichment and localize all along the lateral membrane (Choi et al., 2013). Rap1 loss leads to total loss of Cno recruitment to the plasma membrane and mimics effects of Cno loss on Arm and Baz localization (Choi et al., 2013). Surprisingly, deleting both RA domains leads to an intermediate phenotype. Cno TCJ enrichment is lost and Cno is not as tightly restricted to apical SAJs, and Arm and Baz enrichment at nascent junctions is reduced (Bonello et al., 2018; Perez-Vale et al., 2021).

We thus examined CnoΔRA1 localization and function during AJ establishment. In *cno*Δ*RA1* maternal–zygotic mutants we were surprised to find that CnoΔRA1 was largely absent from the membrane (compare Fig. 4A″ versus D″, B″ versus E″, and C″ versus F″). Occasional Cno puncta accumulated along lateral membranes, both at the level of SAJs (Fig. 4F–F″) and mislocalized to basal junctions (Fig. 4G–G″). This is clearest in MIPs (Fig. 4E–E″). Loss of membrane localization was consistent; in embryos stained in the same experiments 14 of 14 cellularizing embryos had little or no membrane localization of CnoΔRA1, whereas CnoΔRA1 localized to AJs in 17 of 17 post-cellularization embryos. This contrasts with CnoΔRA, lacking both RA domains, which remains enriched in membrane-proximal puncta (Fig. 4K,K′; Perez-Vale et al., 2021), though it loses tight SAJ restriction (Fig. 4J–J″). *cno*Δ*RA1* mutants also failed to enrich Arm apically (Fig. 4D,D′,E,E′, green arrowheads), though some enrichment at basal junctions remained (Fig. 4D,D′,E,E′, red arrowheads). Baz was also no longer restricted to nascent apical junctions (Fig. 4I), although it continued to associate with lateral membranes (Fig. 4H). Thus, CnoΔRA1 cannot support initial junctional polarization and has strongly reduced membrane localization; this suggests at this stage CnoΔRA1 is even less functional than CnoΔRA, in which Cno localization to nascent SAJs is reduced but not eliminated (Perez-Vale et al., 2021). Perhaps RA2 blocks Cno localization at this stage in the absence of RA1. Intriguingly, as gastrulation began (stage 6), CnoΔRA1 returned to apical AJs (Fig. 4L–L″). CnoΔRA also returns to AJs at this stage, as does wild-type Cno in *Rap1* mutants (Bonello et al., 2018).

### RA1 deletion destabilizes AJs under elevated tension and alters junctional protein planar polarity

The morphogenetic movements of gastrulation, germband extension and dorsal closure all require Cno to stabilize AJs under elevated force as cells change shape. In its absence, AJs under elevated tension, like those at aligned anterior–posterior (AP) borders or multicellular junctions, separate and morphogenetic movements fail. Since *cno*Δ*RA* is defective in all these Cno functions (Perez-Vale et al., 2021), we tested whether morphogenesis, AJ stability and junctional protein planar polarity require RA1.

**Fig. 5.**
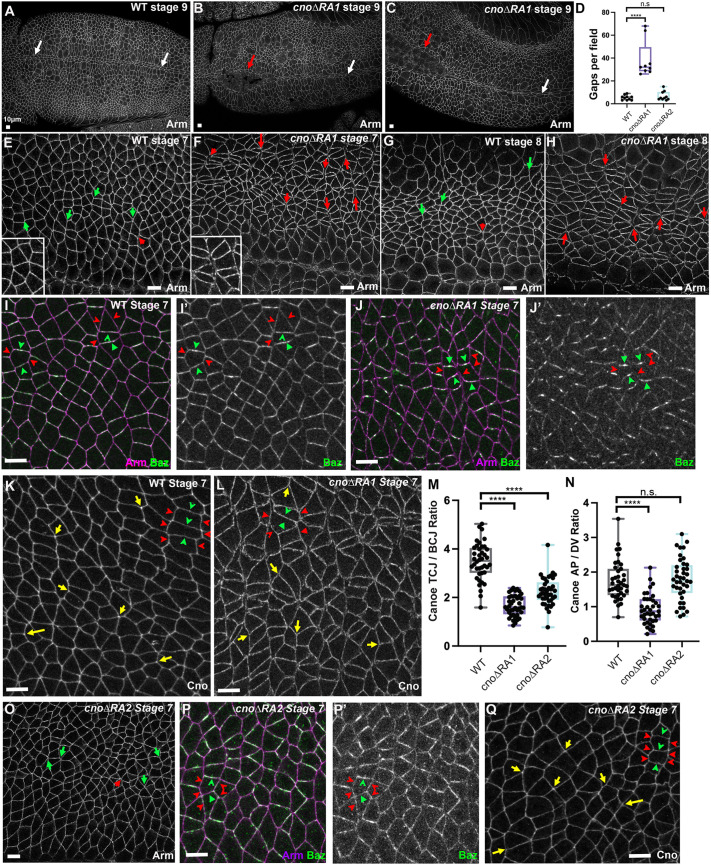
**The Cno RA1 domain is important for reinforcing AJs under tension and for Cno enrichment at these junctions.** Immunofluorescence images of embryos, anterior left, dorsal up; stage, genotype and antigens indicated. (A) Wild-type (WT) embryo. Ventral furrow is completely closed (arrows). (B,C) In many *cno*Δ*RA1* mutants, the furrow has not fully closed (red arrows). White arrows indicate closed ventral furrow. (D) Quantification of AJ gaps per field of view at stages 7 and 8. *n*=9–10 embryos analyzed per genotype. (E,G) Wild-type embryos. At stages 7 and 8, very few AJ gaps are seen (green arrows, no gap; red arrows, gap). (F,H) *cno*Δ*RA1* mutants have numerous gaps at aligned AP borders and rosette centers (red arrows). Insets in E and F show enlarged regions illustrating this. (I,I′) Wild-type embryo. Baz is subtly enriched at DV borders (green arrowheads) as compared to AP borders (red arrowheads) but surrounds each cell. (J) *cno*Δ*RA1.* Baz is substantially reduced at AP borders (red arrowheads) and often confined to the center of DV borders (green arrowheads). (K) Wild-type embryo. Cno is enriched at many TCJs (yellow arrows) and subtly enriched at AP borders (red arrowheads) as compared to DV borders (green arrowheads). (L) *cno*Δ*RA1.* TCJ and AP border enrichment are reduced (arrows and arrowheads as in K). (M,N) Quantification of Cno enrichment at TCJs (M) and AP borders (N). *n*=40 TCJ or aligned AP borders from four embryos per genotype. (O) *cno*Δ*RA2*. Few gaps are seen in AJs (green arrows, no gap; red arrow, gap). (P,P′) *cno*Δ*RA2*. Baz localization is relatively normal (green and red arrowheads as in E). (Q) *cno*Δ*RA2*. Some TCJ enrichment remains (yellow arrows) and AP border enrichment is unchanged (red arrowheads, AP borders; green arrowheads, DV borders). Box and whisker plots in D,M,N show the median (line), 25th–75th percentiles (box) and 5th–95th percentiles (whiskers). ****P<0.0001; n.s., not significant (ANOVA and Brown–Forsythe test). All scale bars: 10 μm.

Gastrulation begins with mesoderm invagination. In wild-type embryos the ectoderm seals at the midline, leaving no gap (Fig. 5A). *cno* null mutants exhibit fully penetrant defects in this, with mesodermal cells remaining on the surface and the furrow not fully closing (Sawyer et al., 2009). In *cno*Δ*RA1* mutants, 25 of 29 stage 7–9 embryos had defects in mesoderm invagination, ranging from mild defects (Fig. 5B; 10/29 embryos) to more wide-open ventral furrows (Fig. 5C; 15/29 embryos). This was similar to the defect frequency in *cno*Δ*RA* mutants (Perez-Vale et al., 2021).

Germband extension is driven in part by reciprocal planar polarization of AJ and cytoskeletal proteins, with actin and myosin enriched on AP borders, and AJ proteins and Baz enriched on dorsal–ventral (DV) borders. Junctional myosin contractility then shrinks AP borders and rearranges cells. We first examined whether RA1 is essential to reinforce AJs under tension. As wild-type germband extension starts at stage 7, Arm localizes all around cells, with few apical gaps in AJs (Fig. 5E, compare green arrows versus red arrows). In contrast, in *cno*Δ*RA1* mutants apical gaps appeared at many aligned AP borders and multicellular junctions (Fig. 5F, red arrows; compare insets in Fig. 5E,F), like those observed in *cno*Δ*RA* mutants (Perez-Vale et al., 2021). Similar gaps were seen at stage 8 (see red arrows in Fig. 5G,H), as cells in mitotic domain 11 rounded up to divide. Quantification verified a substantial increase in gap number in *cno*Δ*RA1* mutants (Fig. 5D; *P*<0.001; one-way ANOVA with Brown–Forsythe test), with gap frequency similar to that in *cno*Δ*RA* mutants (Perez-Vale et al., 2021). Cno also restrains Baz planar polarization. In wild-type embryos, Baz accumulates all around the cell, with ∼2-fold enrichment on DV borders compared to AP borders (Fig. 5I,I′, green arrowheads versus red arrowheads). In the absence of Cno, levels of Baz are reduced on AP borders and particularly enriched in the center of DV borders, elevating Baz planar polarization (Sawyer et al., 2011). *cno*Δ*RA1* mutants also had strong alterations in Baz localization, with very strong Baz reduction on many AP borders (Fig. 5J,J′, red arrowheads), and Baz often restricted to the center of DV borders (Fig. 5J,J′, green arrowheads). Again, this was similar to both *cno* null (Sawyer et al., 2011) and *cno*Δ*RA* mutants (Perez-Vale et al., 2021). Because *cnoRA2RA2* mutants had cuticle defects similar to those of *cno*Δ*RA1* mutants, we examined protein localization and junctional phenotypes. *cnoRA2RA2* phenotypes were similar to those of *cno*Δ*RA1* mutants (Fig. S3A–A‴). In contrast, CnoRA1RA1 was enriched at TCJs and aligned AP borders, and Arm localization to AJs appeared normal in *cnoRA1RA1* mutants (Fig. S3C–D″). Thus, RA1 is crucial for Cno to reinforce AJs under elevated tension and to restrain Baz planar polarity – in these ways the *cno*Δ*RA1* mutant closely resembles mutants lacking both RA domains (Perez-Vale et al., 2021). Furthermore, replacing the missing RA1 domain with a copy of RA2 does not rescue function.

### RA1 is important for Cno enrichment at AJs under elevated tension

Cno protein is normally enriched at TCJs (Fig. 5K, yellow arrows; quantified in Fig. 5M), and is subtly enriched at AP borders (Fig. 5K, red arrowheads versus green arrowheads; quantified in Fig. 5N), the same places where junctions apically separate in *cno* mutants (Perez-Vale et al., 2021). We next asked whether RA1 is required for this enrichment. Inspection suggested reduced CnoΔRA1 enrichment at TCJs (Fig. 5L, yellow arrows) and loss of enrichment at AP borders (Fig. 5L, red arrowheads versus green arrowheads). We quantified the ratio of Cno at TCJs and bicellular junctions, revealing that deleting RA1 strongly reduces but does not eliminate TCJ enrichment (Fig. 5M). Quantification also verified loss of Cno enrichment at AP borders in *cno*Δ*RA1* mutants (Fig. 5N). Intriguingly, these phenotypes were similar to but not as strong as those of *cno*Δ*RA* mutants, which completely lose Cno enrichment at TCJs and in which Cno enrichment is reversed relative to wild-type enrichment, with elevation at DV borders (Perez-Vale et al., 2021).

### The RA2 domain is not essential for AJ stabilization

In parallel, we examined the role of RA2 in stabilizing AJs under elevated tension, Baz planar polarization and Cno localization to AJs under tension. *cno*Δ*RA2* mutants had no significant increase in apical gaps in AJs relative to wild-type embryos (Fig. 5O, quantified in 5D). Baz continued to localize to both AP and DV borders, without the extreme planar polarity seen in *cno*Δ*RA1* mutants (compare Fig. 5P,P′ and J,J′). Whereas CnoΔRA2 protein was enriched at TCJs (Fig. 5Q, yellow arrows), quantification revealed that average enrichment was reduced relative to wild-type enrichment, though not to the extent seen in *cno*Δ*RA1* mutants (Fig. 5M). Cno enrichment at aligned AP borders in *cno*Δ*RA2* mutants was similar to that in wild-type embryos (Fig. 5N). Thus, RA2 is not essential for most embryonic functions of Cno, though it might enhance enrichment at some AJs under elevated tension.

### RA1 is essential for late morphogenesis and to maintain epidermal integrity

Cno is also essential for later morphogenetic movements, including dorsal closure and head involution (Boettner et al., 2003; Jürgens et al., 1984). In *cno* maternal–zygotic null mutants both events fail, and cells in the ventral epidermis have difficulty returning to columnar architecture after rounding up to divide, ultimately leading to holes in the ventral epidermis as observed in cuticles. We thus examined whether RA1 was important for these Cno functions.

During wild-type stages 9 and 10, ectodermal cells divide into three groups. Dorsal ectodermal cells complete cell division in stage 8 and resumed columnar architecture. Cells in the lateral and ventral ectoderm divide during stages 9 and 10, respectively, and need to remodel AJs as they round up for mitosis and then resume columnar architecture. Simultaneously, ∼30% of these cells apically constrict and move inward to become neural stem cells. These twin challenges mean that the ventral epidermis is more sensitive to disruptions in adhesion or AJ–cytoskeletal linkage.

**Fig. 6.**
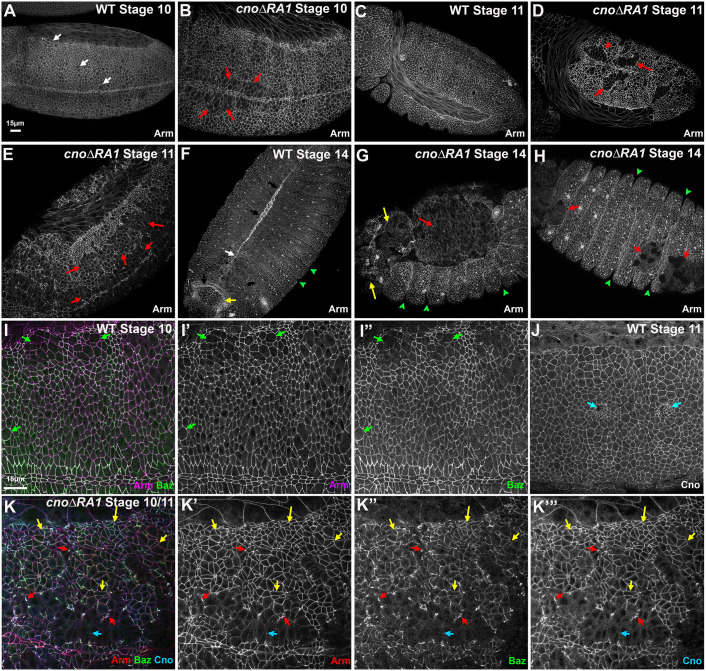
**The Cno RA1 domain is important for dorsal closure, head involution and epidermal integrity.** Immunofluorescence images of embryos, anterior left and dorsal up unless stated; stage, genotype and antigens indicated. (A,B) Stage 10. (A) Wild-type (WT) embryo. Some cells are rounded up to divide (white arrows), but they rapidly return to columnar architecture. (B) *cno*Δ*RA1.* Groups of cells near the ventral midline fail to resume columnar architecture (red arrows). (C–E) Stage 11. (C) Wild-type embryo. (D,E) *cno*Δ*RA1.* Failure to resume columnar architecture becomes more apparent (red arrows). (F–H) Stage 14. (F) Ventral view of a wild-type embryo. Dorsal closure is completed (white arrow), head involution is underway (yellow arrow) and segmental grooves are shallow (green arrowheads). (G) *cno*Δ*RA1.* Dorsal closure failed, exposing underlying tissues (red arrow). There are gaps in the head epidermis (yellow arrows) and deep segmental grooves remain (green arrowheads). (H) Ventral view, *cno*Δ*RA1.* Holes in the epidermis (red arrows) and deep segmental grooves (green arrowheads) are observed. (I–J) Closeups, wild-type stage 10 (I–I″) and 11 (J). Dividing cells (green arrows) and forming tracheal pits (cyan arrows) are observed. Arm, Baz and Cno remain enriched at AJs. (K–K‴) Stage 10/11 *cno*Δ*RA1* mutant. Cells that retained columnar architecture retain junctional Arm, Baz and CnoΔRA1 (yellow arrows). However, in some cells AJs are fragmented (red arrows) and in less epithelial regions Arm, Baz and Cno are strongly reduced (cyan arrows). Scale bar in A applies to A–H; scale bar in I applies to I–K‴. Images are representative of more than 30 embryos from six experiments.

By wild-type stage 10, most cells completed division, although a subset near the ventral midline remain rounded up in mitosis (Fig. 6A, white arrows). Cells rapidly resume columnar architecture after division. In contrast, in *cno*Δ*RA1* mutants, cells near the ventral midline failed to return to columnar architecture (Fig. 6B, red arrows). By stage 11 in wild-type embryos (Fig. 6C), cell division was largely complete, with cell shape changes driving head segmentation and tracheal pit invagination. In *cno*Δ*RA1* mutants at stage 11, groups of ventral cells that had failed to return to a columnar architecture remained, creating epithelial gaps – the severity of this phenotype varied from moderate (Fig. 6D) to more severe (Fig. 6E). We also examined AJ protein localization. At wild-type stages 10 and 11, both Arm (Fig. 6I,I′) and Cno (Fig. 6J) were strongly enriched at AJs, with modestly reduced accumulation in cells rounded up to divide. Baz also localized to AJs all around cells (Fig. 6I″). In *cno*Δ*RA1* mutants, epithelial cells retaining columnar architecture retained junctional Arm and Cno (Fig. 6K,K′,K‴, yellow arrows), whereas cells that had failed to resume columnar architecture had strongly reduced levels at AJs (Fig. 6K,K′,K‴, cyan arrow). Baz localization to AJs was reduced in most columnar cells (Fig. 6K,K″, yellow arrows), and AJs were fragmented where columnar and non-columnar cells met, which perhaps represent places where AJ remodeling is maximal. Junctional fragments accumulated all three AJ proteins (Fig. 6K–K‴, red arrows). All are defects seen in our strongest *cno* mutants, including *cno*Δ*RA* (Manning et al., 2019; Perez-Vale et al., 2021). *cnoRA2RA2* mutants had similar defects (Fig. S3B–B‴).

After germband retraction, two groups of cells begin complex collective movements. In dorsal closure, lateral epidermal sheets extend dorsally and zip together, encasing embryos in epidermis (Fig. 6F, white arrow), while more anterior cells complete head involution (Fig. 6F, yellow arrow). In *cno*Δ*RA1* mutants, both dorsal closure and head involution failed, which is consistent with the cuticle phenotypes. Dorsal closure was not complete before amnioserosal cells apoptosed, leaving the dorsal side open (Fig. 6G, red arrow); gaps in epidermal coverage of the head were also apparent (Fig. 6G, yellow arrows). Ventrally, holes remained in the epidermis (Fig. 6H, red arrows), consistent with earlier defects (Fig. 6D,E). Persistent deep segmental grooves remained (Fig. 6G,H green arrows) – another *cno* null mutant phenotype. Putting together the phenotypes, these data reveal that RA1 is necessary for all of the roles played by Cno in embryonic morphogenesis, and it cannot be replaced by a second copy of RA2, despite the shared ability of these domains to bind Rap1. RA2 only plays minor roles in Cno recruitment to AJs under tension. This rules out many possible mechanistic hypotheses for function.

### Sensitized assays reveal that CnoΔRA2 does not provide full Cno function

**Fig. 7.**
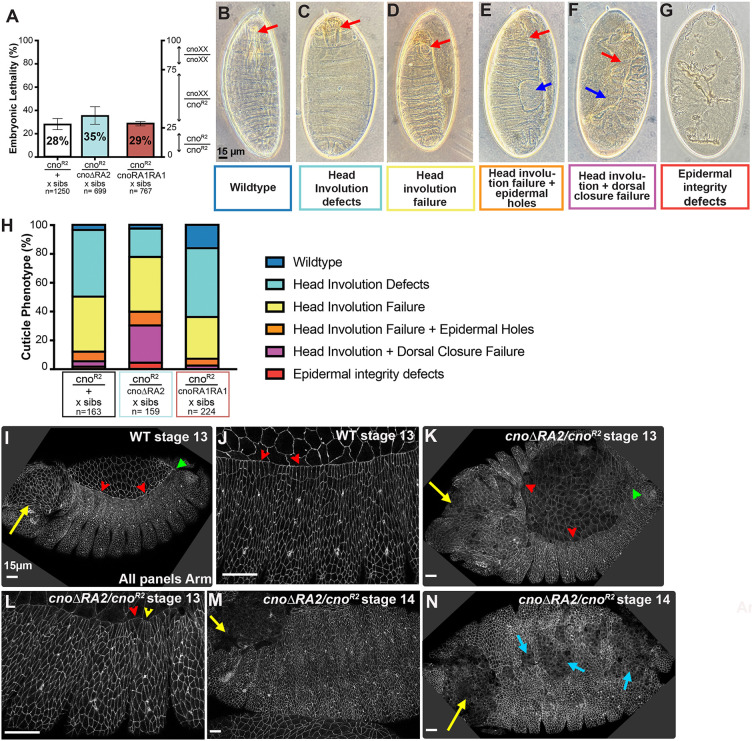
**A sensitized assay reveals that CnoΔRA2 does not provide fully wild-type function in embryonic morphogenesis.** (A) Quantification of embryonic lethality for the indicated genotypes (sibs, siblings). Right-hand axis shows expected distribution of genotypes at Mendelian ratios. Data are presented as mean±s.d. of three to five experiments. (B–G) Embryonic cuticles illustrating range of severity of phenotypes (images also used in Fig. 3G–L). Scale bar in B applies to B–G. (H) Stacked bar graphs illustrating cuticle defects in the indicated genotypes. Mutants were scored according to the phenotype categories illustrated in B–G. Most *cno^R2^* zygotic mutants only have defects in head involution. Progeny of *cno^R2^/cno*Δ*RA2* parents have more severe defects, with frequent dorsal closure failure. In contrast, progeny of *cno^R2^/cnoRA1RA1* parents have defects indistinguishable from those of *cno^R2^/+* parents. (I–N) Immunofluorescence images of embryos, anterior left; stages and antigen indicated. Embryos labeled *cno*Δ*RA2/cno^R2^* are progeny of *cno*Δ*RA2/cno^R2^* parents – genotype was not determined. (I,J) Wild-type (WT) embryo at stage 13. (I) Dorsal closure (red arrowheads) and head involution (yellow arrow) are proceeding, with zipping beginning at canthi (green arrow). (J) The leading edge is straight, and cell shapes are relatively uniform (red arrowheads). (K–N) *cno*Δ*RA2/cno^R2^* at stage 13 and 14, as indicated. The leading edge is wavy (K, red arrowheads), zipping is slowed (K, green arrowhead), leading edge cell shapes are less uniform (L, arrowheads), head involution fails (M and N, yellow arrows) and ventral epidermal holes are sometimes present (N, cyan arrows). Images are representative of 13 embryos from three experiments. Scale bars (I–N): 15 μm.

The viability and fertility of the *cno*Δ*RA2* mutant was surprising, given the conservation of RA2 over ∼600 million years of animal evolution. Some other mutants, including *cno*Δ*PDZ* and *cno*Δ*FAB,* are also viable (Perez-Vale et al., 2021), despite similar conservation of those protein domains or regions. However, previous sensitized assays revealed neither of these mutants retains full wild-type function. To conduct sensitized assays, we reduced the maternal and zygotic dose of mutant protein by making mothers and fathers trans-heterozygous for the mutant of interest and our null allele, *cno^R2^*, and examining progeny. Our control involved parents heterozygous for a wild-type chromosome and *cno^R2^* (*cno^R2^*/+). *cno^R2^* is zygotically embryonic lethal, and thus we expected 25% embryonic lethality. In parallel we crossed *cno^R2^*/*cno*Δ*RA2* parents. This modestly elevated lethality (35% lethality versus 28% lethality in the control; Fig. 7A), suggesting that some *cno^R2^*/*cno*Δ*RA2* embryos die.

We next examined morphogenesis using cuticle phenotypes (Fig. 7B–G). The strong maternal Cno contribution allows most events of morphogenesis go to completion in *cno^R2^* zygotic mutants, so most embryos only exhibit defects in head involution (89%; as depicted in Fig. 7C,D, quantified in Fig. 7H), whereas dorsal closure generally is completed (4% of embryos fail). In contrast, cuticle phenotypes of the progeny of *cno^R2^*/*cno*Δ*RA2* parents were substantially enhanced, with 31% of dead embryos exhibiting dorsal closure failure (as depicted in Fig. 7F, quantified in 7H; *n*=159). In contrast, *cnoRA1RA1* behaved essentially like a wild-type allele in this assay, with 29% embryonic lethality (Fig. 7A) and morphogenesis defects similar to the *cno^R2^*/+ control: 92% only had defects in head involution and only 3% had defects in dorsal closure (Fig. 7H; *n*=224).

Consistent with the cuticle phenotypes, we observed defects in dorsal closure and head involution in progeny of *cno^R2^*/*cno*Δ*RA2* parents (Fig. 7I–N), including defects in zippering, a wavy leading edge (compare Fig. 7I and K, red arrowheads), and uneven leading edge cell shapes (compare Fig. 7J and L) with hyperconstricted (Fig. 7L, yellow arrowhead) or splayed open cells (Fig. 7L, red arrowhead). Holes were seen in the head epidermis (compare Fig. 7I with K, M and N, yellow arrows). Thus, CnoΔRA2 does not confer fully wild-type function, whereas RA1 functionally replaces RA2 in this assay, providing further mechanistic insights.

### The Cno RA2 domain is required for patterning the developing eye

In addition to its roles in embryonic morphogenesis, Cno is also important for the intricate cell shape changes and rearrangements transforming the uniform epithelium of the eye imaginal disc into the stereotyped arrangement of epithelial cell types in the ∼750 ommatidia. *cno* was first identified because weak alleles alter the stereotyped arrangement of ommatidial cells (Gaengel and Mlodzik, 2003; Matsuo et al., 1997; Matsuo et al., 1999), whereas complete loss of Cno function dramatically disrupts epithelial architecture (Walther et al., 2018).

**Fig. 8.**
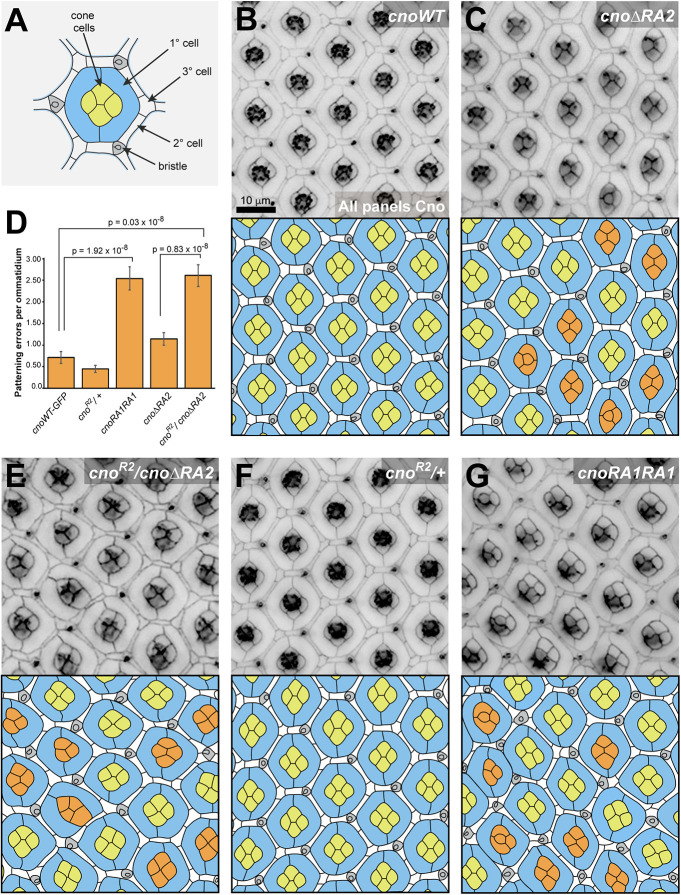
**The Cno RA2 domain is essential for morphogenesis in the pupal eye.** (A) Diagram of a pupal eye ommatidium 40 h after puparium formation. (B,C) Immunofluorescence images showing regions of *cno-wt-GFP* (B) and cnoΔ*RA2* (C) homozygote eyes 40 h after puparium formation, with tracings of images below. Incorrectly organized cone cells are colored orange in the tracings. (D) Patterning errors for all genotypes quantified as average number of patterning errors per ommatidium. Data are presented as mean±s.e.m. of *n*=76–110 ommatidia from three experiments. Statistical significance determined using two-tailed two-sample with unequal variance Student's *t*-tests. (E–G) Regions of *cno^R2^*/*cno*Δ*RA2* (E), *cno^R2^*/+ (F) and *cnoRA1RA1* (G) eyes 40 h after puparium formation, with tracings of images below. Incorrectly organized cone cells are colored orange in the tracings. GFP-tagged Cno was detected in B,C,E and G, and endogenous Cno was detected in F. Scale bar in B applies to B,C,E–G.

Because the *cno*Δ*RA1* mutation is lethal, we could not examine eye development in this genotype. To further explore RA2 function, we examined whether *cno*Δ*RA2* mutants have defects in eye development, looking 40 h after pupal development began. Each nascent ommatidium has a stereotyped arrangement of epithelial cell types, each with characteristic shapes (Fig. 8A,B; Johnson, 2021). At the center are four cone cells surrounded by two primary (1°) pigment cells. These are separated from cells of neighboring ommatidia by a lattice of rectangular secondary (2°) pigment cells, hexagonal tertiary (3°) pigment cells and mechanosensory bristles. Deleting RA2 introduced numerous patterning errors, most notably in cone cell organization (Fig. 8C). During development cone cells are recruited to ommatidia with the anterior and posterior cells initially in direct contact and the dorsal and ventral pair occluded. A T1–T2–T3 junction exchange normally reorients this (Fig. 8A,B), but the AP cone cells remained in contact in many *cno*Δ*RA2* ommatidia. We also observed clusters where one of the four cone cells failed to maintain contact with its neighboring 1° cell (Fig. 8C). These defects, as well as minor defects in lattice cell organization in *cno*Δ*RA2* ommatidia, were quantified using the ommatidial mis-patterning score (Fig. 8D; Table S1; Johnson and Cagan, 2009). Molecular control of the cone cell T1–T2–T3 transition is not well understood, with limited studies suggesting adhesive rather than cytoskeletal changes as principal drivers (Blackie et al., 2021; Grillo-Hill and Wolff, 2009). Our data highlight the importance of Cno in this process. The cone cell defects in *cno*Δ*RA2* retinas were amplified in *cno^R2^*/*cno*Δ*RA2* trans-heterozygotes, where cone cell numbers were occasionally reduced and the lattice was more severely mis-patterned (Fig. 8E; *cno^R2^*/+ retinas were not mis-patterned, Fig. 8F). We also assessed whether replacing the missing RA2 domain with a second RA1 domain rescued these defects. Surprisingly, cone cell and lattice cell defects were prevalent in *cnoRA1RA1* homozygotes (Fig. 8G), emphasizing an important requirement for the RA2 domain of Cno in pupal eye morphogenesis. Thus, RA2 also has specific mechanistic roles in Cno function.

## DISCUSSION

Cno and afadin are key elements of the protein network linking cell–cell AJs to the actomyosin cytoskeleton, allowing cells to change shape and move without tissue disruption. Both are Rap1 effectors and are thought to be activated via Rap1 binding. However, unlike many Ras-family GTPase effectors, Cno and afadin have two tandem RA domains. Deleting both nearly eliminates Cno function, but major questions remained about the mechanistic functions of the individual RA domains. We combined biochemical, genetic and cell biological analyses to test different mechanistic hypotheses.

### RA1 and RA2 have dramatically different roles in Cno function in morphogenesis

Cno and afadin are among the effectors of Rap1, and data suggest that Rap1 activates them to carry out their roles (Boettner et al., 2003; Bonello et al., 2018; Hiremath et al., 2023; Pannekoek et al., 2020; Perez-Vale et al., 2023; Sawyer et al., 2009; Walther et al., 2018). Deleting both RA domains has been found to severely diminish Cno function in multiple events of embryonic morphogenesis (Perez-Vale et al., 2021), but the mechanisms by which the two RA domains regulate Cno function remained a key question. Different hypotheses predicted that they might be redundant in function or might have different roles. Our new mutants provide clear answers. RA1 is essential for all Cno functions in embryonic morphogenesis and plays an important role in Cno recruitment to AJs under tension. In contrast, RA2 is dispensable for viability and fertility. However, RA2 has accessory roles, helping explain its evolutionary retention. It is important for high-fidelity assembly of ommatidia in compound eyes, and in this role cannot be replaced by RA1. Furthermore, in sensitized assays in embryos RA2 is required to provide fully wild-type function. Intriguingly, neither Cno RA domain is essential for recruitment to AJs after gastrulation onset, whereas both contribute to mechanosensitive recruitment to AJs under elevated tension. We also tested the hypothesis that the two RA domains are equivalent, with the N-terminal position of RA1 being crucial. However, replacing RA1 with a second copy of RA2 (*cnoRA2RA2*) did not restore function, disproving this hypothesis.

These differential functions contrast with the roles of the two RA domains in PLCε where both are essential. RA2 mediates PLCε activation by Ras, Rap1 and their upstream activators, as well as PLCε recruitment to the plasma membrane (Bunney et al., 2006; Kelley et al., 2001; Song et al., 2002), while RA1 plays a stabilizing structural role (Rugema et al., 2020).

### RA1 and RA2 bind Rap1 with similar affinities

Previous biochemical assays support the hypothesis that the Cno/afadin RA domains differ in Rap1 binding, potentially causing differences in function. Multiple groups have assessed activated Rap1 binding to afadin RA1, measuring *K*_d_ values of 0.2–3 µM (Linnemann et al., 1999; Smith et al., 2017; Wohlgemuth et al., 2005), and the results of less quantitative yeast two-hybrid assays support this (Boettner et al., 2003). In contrast, existing data on RA2 are conflicting – biochemical assays have found no measurable binding between afadin RA2 and activated Rap1 (Wohlgemuth et al., 2005), whereas the results of yeast two-hybrid assays suggest weaker but detectable binding of Cno RA2 and Rap1 (Boettner et al., 2003). This issue has been complicated by the recent realization that RA1 and likely RA2 have additional N-terminal α-helices (αN helices). The αN helix has been found to boost RA1 binding to Rap1 4-fold (Smith et al., 2017). In a previous study the αN helix was absent from the afadin RA2 construct used to measure binding (Wohlgemuth et al., 2005), and was partially truncated in Cno RA2 constructs used in two-hybrid assays (Boettner et al., 2003).

We thus examined binding of Cno RA1 or Cno RA2 to activated Rap1, using constructs retaining the αN helices. To our surprise, both SEC-MALS and ITC revealed that each RA domain binds activated Rap1, with similar affinities. Furthermore, the fact that both RA domains remain associated with Rap1 through the SEC-MALS run suggests formation of a relatively stable complex. This disproves the hypothesis that the dramatic functional differences observed *in vivo* are simply due to only one RA domain being able to bind Rap1, or to substantial differences in Rap1 binding affinity. Given the similar affinities, it is truly surprising the two RA domains are not at least somewhat redundant in function, but our functional data support this. Furthermore, RA1 cannot be replaced by RA2, and, in the eye, RA2 cannot be replaced by RA1. This leaves alternate mechanistic hypotheses. The stronger evolutionary conservation of RA1 between mammals and *Drosophila* (74% amino acid identity for RA1 versus 54% amino acid identity for RA2) may suggest that RA1 has additional roles. Perhaps RA1 uses a conserved protein interface outside the Rap1 binding region to bind other domains of Cno/afadin or other protein partners. In the future, identifying what might bind RA1 and not RA2, and vice versa, could provide insights.

### By what mechanism does Rap1 ‘activate’ Cno?

We have known for 20 years that Cno is a Rap1 effector during embryogenesis (Boettner et al., 2003). Cno and Rap1 share virtually identical roles in initial AJ positioning and mesoderm apical constriction (Choi et al., 2013; Sawyer et al., 2009; Spahn et al., 2012). Rap1 appears to have additional effectors during later morphogenesis, but Cno continues to be one of its effectors in germband extension, dorsal closure and epidermal integrity (Boettner et al., 2003; Perez-Vale et al., 2023), as well as post-embryonic eye development (Walther et al., 2018). Since Cno RA1 and RA2 bind Rap1 with high affinity, how does Rap1 binding ‘activate’ Cno?

We initially considered two roles: Rap1 binding mediates plasma membrane recruitment and assembly into AJs, or Rap1 binding triggers conformational changes that ‘open up’ a closed conformation of Cno, allowing it to interact with protein partners. We tested these hypotheses. The combined RA domains are important for initial polarized recruitment of Cno to nascent AJs as they form during cellularization (Perez-Vale et al., 2021), and Rap1 is also essential for this. However, CnoΔRA returns to apical AJs as gastrulation starts (Perez-Vale et al., 2021), and wild-type Cno returns to apical AJs at this time in *Rap1* mutants (Perez-Vale et al., 2023). Thus, AJ recruitment occurs by multiple means, and this is not the sole role of Rap1. Our data also suggest that Rap1 influences Cno localization and function in ways that do not solely rely on the RA domains. Cno is entirely absent from the plasma membrane during AJ assembly in *Rap1* mutants (Sawyer et al., 2009), whereas CnoΔRA protein remains associated with the lateral membrane but loses apical polarization (Perez-Vale et al., 2021). This suggests RA domain-independent effects of Rap1 on the initial membrane recruitment of Cno. Even more puzzling, our new data reveal that initial CnoΔRA1 plasma membrane recruitment is reduced relative to that of CnoΔRA, suggesting that at this stage RA2 might antagonize Rap1-independent membrane recruitment. The multivalent recruitment of Cno to AJs is further illustrated by the fact that none of the *cno* mutants thus far tested – together deleting both RA domains (Perez-Vale et al., 2021), the Dilute domain (McParland et al., 2024), the PDZ domain (Perez-Vale et al., 2021) or the F-actin-binding (FAB) region (Perez-Vale et al., 2021) – abolish gastrulation-stage AJ recruitment of Cno. This recruitment is also Rap1 independent (Bonello et al., 2018). Thus, AJ recruitment is remarkably robust and cannot be the only role of Rap1.

In some proteins activated by small GTPase binding, like formins, GTPase binding triggers conformational changes releasing intramolecular inhibition. This was originally a favored hypothesis. However, in some versions of this model, CnoΔRA would be constitutively active, which is something we did not observe. Furthermore, this idea suggests that the N-terminal folded domains have a quaternary structure that is altered by Rap1 binding. However, deleting the RA2, Dilute or PDZ domains does not affect viability (these data; Perez-Vale et al., 2021; McParland et al., 2024), seriously calling this model into question. Some versions of this model remain plausible, as RA1 might bind other regions of Cno that we have yet to mutate, such as the Forkhead-associated (FHA) domain or the IDR. Future mutational analysis, including making versions of RA1 that cannot bind Rap1 but retain other putative protein interaction interfaces, may provide insights.

## MATERIALS AND METHODS

### Structure prediction and structure analysis

The *Drosophila* Cno RA domain–Rap1 complex structure prediction models were computed using ColabFold v1.5.5 running AlphaFold2-multimer (Jumper et al., 2021; Mirdita et al., 2022). Amino acid sequence inputs were Canoe RA1 residues 1–137 and Rap1 residues 1–184, and Canoe RA2 residues 222–350 and Rap1 residues 1–184. The mouse afadin RA1–human Ras-GMPPNP complex was obtained from PDB 6AMB (https://www.rcsb.org/structure/6AMB; Smith et al., 2017). Structures were aligned using the align command in PyMOL (Schrodinger). Protein sequence alignments were generated manually based on output from the PyMOL structural alignments.

### Cloning and purification of Cno RA domains and Rap1G12V GTPase

DNA encoding the *Drosophila melanogaster* Cno RA1 and RA2 domains (amino acids 1–137 and 222–350, respectively) was generated using PCR (RA1 forward primer, 5′-CGCTGTTCGCAT ATGTCACATGATAAGAAGATGTTGGATCGCGAGGCAGTACGC-3′; RA1 reverse primer, 5′-CACTCGTTCGAATTCTTAGGGAGTGGTCTTTTGGTCTATGTTCTTGAGCAG-3′; RA2 forward primer, 5′-GCGTGTTCGCATATGACCCGATCGATCTCAAACCCGGAGGCCGTG-3′; RA2 reverse primer, 5′-GAGTCGTTCGAATTCTTATGAGTCCGCCGGACGTCTCCGCACATG-3′) and sub-cloned into pET28 (Millipore Sigma) using Nde1 and EcoR1 restriction enzymes and T4 DNA ligase (New England Biolabs). Similarly, DNA encoding the *D. melanogaster* GTPase Rap1G12V (amino acids 1–180) was generated using PCR and sub-cloned into pET28 using Nde1 and EcoR1 restriction enzymes and T4 DNA ligase (Rap1 forward primer, 5′-CGCTGTTCGCATATGCGTGAGTACAAAATCGTGGTCCTTGG-3′; Rap1 reverse primer, 5′-CACTCGTTCGAATTCTCATAGGGACTTTTTCGGCTTCTTCTG-3′). The plasmids were transformed into *Escherichia coli* BL21 DE3 pLysS cells and grown to an optical density at 600 nm of 0.8 in medium containing 50 µg/l kanamycin at 37°C. The temperature was lowered to 20°C, and protein expression was induced with 100 µM IPTG for 16 h. Cells were harvested by centrifugation, resuspended in buffer A [25 mM Tris-HCl pH 8.5, 300 mM NaCl, 10 mM imidazole and 0.1% β-mercaptoethanol (β-ME)] at 4°C and lysed by sonication (the Rap1G12V buffer A was supplemented with 1 mM MgCl_2_ and 5% glycerol). Phenylmethylsulfonyl fluoride was added to 1 mM final concentration. Cells debris was pelleted by centrifugation at 17,000 ***g*** for 1 h, and the supernatant was loaded onto a 10 ml Ni^2+^-NTA column (Qiagen). The column was washed with 1 l buffer A, and the protein batch eluted with 100 ml buffer B (buffer A supplemented with 290 mM imidazole). CaCl_2_ was added to 1 mM final concentration. RA1 domain protein received 15 µl (3.6 mg/ml) of bovine α-thrombin (Prolytix) and was incubated at 4°C for 36 h to proteolytically cleave off the N-terminal His_6_ tag. Rap1G12V protein received 40 µl (9.6 mg/ml) of bovine α-thrombin to proteolytically cleave off the N-terminal His_6_ tag and was incubated at 4°C for 4 days. Thrombin-treated RA1 and Rap1G12V batches were dialyzed against 4 l buffer A with no imidazole for 20 h using 3.5K molecular weight cutoff (MWCO) SnakeSkin dialysis tubing (Themo Fisher Scientific). After the dialysis step, protein was filtered consecutively over 0.5 ml benzamadine sepharose (Cytiva) and 10 ml Ni^2+^-NTA column, and the flowthrough was collected. Preparations of RA2, as well as some RA1 preparations, were purified without thrombin treatment or subsequent filtration over benzamidine sepharose or Ni^2+^-NTA resin. All proteins were dialyzed against 2 l buffer C (25 mM HEPES-NaOH pH 7.5, 100 mM NaCl, 5 mM MgCl_2_, 0.1% β-ME) for 5 h, followed by a second dialysis against 2 l buffer C overnight at 4°C. Protein was concentrated in a Millipore Sigma MWCO centrifugal concentrator (3K MWCO for the RA1 and RA2 constructs, 10K MWCO for the Rap1G12V construct), aliquoted and stored at −80°C.

### Nucleotide exchange to generate Rap1G12V-GMPPNP

Alkaline phosphatase (30 U; Millipore Sigma, P-0762) was coupled to agarose beads then washed with 20 mM Tris-HCl pH 7.5. Reaction buffer [40 mM Tris-HCl pH 7.5, 200 mM (NH_4_)_2_SO_4_, 10 µM ZnCl_2_, 5 mM dithiothreitol (DTT)] was added to beads to bring the volume to 100 µl. To the alkaline phosphatase-coupled agarose bead slurry, 10 mg of Rap1G12V and 10 M excess GMPPNP (Jena Bioscience) were added, and the total volume brought to 1 ml using reaction buffer. The mixture was incubated at 4°C with rotation for 3 h. The reaction was centrifuged at 2350 ***g*** for 2 min, the supernatant was divided in half, and each half loaded onto a 2 ml Zeba spin desalting column (Thermo Fisher Scientific) preequilibrated with buffer C. Spin columns were centrifuged at 1000 ***g*** for 2 min to perform buffer exchange. Protein concentration in buffer C was determined, and samples were aliquoted and stored at −80°C.

### Biophysical protein analysis

SEC-MALS runs were performed in buffer C supplemented with 0.2 g/l sodium azide. Samples (100 µl) of individual proteins (200 µM) or RA domain–Rap1G12V-GMPPNP complexes (200 µM complex) were prepared in buffer C and injected onto a Superdex 200 column (Cytiva), run at 0.5 ml/min. The eluate was passed over a DAWN light-scattering instrument (Waters Wyatt Technology) followed by an Optilab dRI detector (Waters Wyatt Technology). Data was processed using the Astra software program (Wyatt Technology) and plotted using DataGraph (Visual Data Tools).

For ITC, Cno RA domain interactions with GMPPNP-loaded Rap1G12V were measured using a MicroCal PEAQ-ITC microcalorimeter (Malvern Panalytical). Protein stock solutions of the respective RA domain and GMPPNP-loaded Rap1G12V were loaded into respective 3.5K MWCO Slide-A-Lyzers (Thermo Fisher Scientific) and dialyzed in a shared 1 l reservoir of buffer C for 1 h, followed by a second 1 l reservoir of buffer C for 2 h, followed by a third 2 l reservoir of buffer C overnight, all at 4°C. Experiments were carried out at 25°C. A 2 µl volume of 150–670 µM GMPPNP-loaded Rap1G12V was automatically injected into a well containing 370 µl of 20–50 µM RA1. A 2 µl volume of 650–1200 µM GMPPNP-loaded Rap1G12V was automatically injected into a well containing 370 µl of 42–80 µM RA2. Twenty injections were performed over the span of 1 h with a reference power of 12 µcal/s, an initial delay of 120 s and a stir speed of 750 rpm. Heats of dilution were determined from control experiments in which GMPPNP-loaded Rap1G12V was titrated into buffer alone. Rap1–RA1 ITC was performed in duplicate (biological). Rap1–RA2 ITC was performed in triplicate (a biological duplicate and a technical duplicate; technical duplicates were experiments 2 and 3). Binding isotherms were fitted to a one-site binding model using MicroCal PEAQ-ITC software (Malvern Panalytical). Rap1–RA1 experiment 1: *K*_d_=4.5 µM, *N*=0.717, Δ*H*=−4381 cal/mol. Rap1–RA1 experiment 2: *K*_d_=5.9 µM, *N*=1.00, Δ*H*=−4791 cal/mol. Rap1–RA2 experiment 1: *K*_d_=8.7 µM, *N*=0.755, Δ*H*=−3073 cal/mol. Rap1–RA2 experiment 2: *K*_d_=8.8 µM, *N*=0.73, Δ*H*=−2178 cal/mol. Rap1–RA2 experiment 3: *K*_d_=7.9 µM, *N*=0.63, Δ*H*=−2718 cal/mol.

### Fly work

Unless otherwise noted, all experiments were performed at 25°C. Flies used as controls, referred to in the text as wild type (WT), were of the *yellow white* genotype [Bloomington Drosophila Stock Center (BDSC), stock 1495]. All Cno constructs are listed in Fig. 3A. *cno*Δ*RA1* germline clones were generated by heat shocking third-instar larvae generated by crossing *cno*Δ*RA1/TM3* virgin females to P{ry[+t7.2]=hsFLP}1, y[1] w[1118]; P{neoFRT}82B P{ovo−D1−18}3R/TM3, ry[*], Sb[1] males in a 37°C water bath for 2 h each on two consecutive days. Then, eclosed virgin females with *hsFLP1; P{neoFRT}82B P{ovo−D1−18}3R/P {neoFRT}82B cnoΔRA1* were collected and subsequently crossed with *cnoΔRA1/TM3 Sb* males. The embryos generated from this cross were analyzed. This same process was performed to create *cnoRA2RA2* germline clones. Allele viability was assessed via a cross to generate mutant flies that are heterozygous for each *cno* allele with the null allele *cno^R2^* (Sawyer et al., 2009).

### Generating mutant *cno* rescue constructs and their ΦC31-mediated integration into the *cno*ΔΔ allele attP site

The *cnoWT-GFP* rescue construct (Perez-Vale et al., 2021) was used to generate new constructs lacking RA1 (*cno*Δ*RA1-GFP*, also referred to here as *cno*Δ*RA1*), lacking RA2 (*cno*Δ*RA2-GFP*, also referred to here as *cno*Δ*RA2*), replacing RA2 with RA1 (*cnoRA1-RA1-GFP*, also referred to here as *cnoRA1RA1*), and replacing RA1 with RA2 (*cnoRA2-RA2-GFP*, also referred to here as *cnoRA2RA2*). To generate the *cno*Δ*RA1-GFP* and *cno*Δ*RA2-GFP* constructs, amino acids 18–136 (RA1) or 211–353 (RA2) of *Drosophila* Cno, respectively, were replaced with an ASGGTS polypeptide linker (DNA sequence 5′-GCTAGCGGCGGCACTAGT-3′, which encodes unique NheI and SpeI restriction enzyme sites) – this was done by Azenta Life Sciences (Waltham, MA, USA). To generate the *cnoRA1-RA1-GFP* construct, DNA encoding RA1 (amino acids 1–135) was amplified using PCR and cloned into the NheI and SpeI sites of the *cno*Δ*RA2-GFP* construct (forward primer, 5′-CGCATCTGAGCTAGCATGTCACATGATAAGAAGATGTTGGATCGCGAGGCAGTACGC-3′; reverse primer, 5′-GCCTAGACTACTAGTGGTCTTTTGGTCTATGTTCTTGAGCAGGAATCGACCTTCGC-3′).

To generate the *cnoRA2-RA2-GFP* construct, DNA encoding RA2 (amino acids 211–353) was amplified using the PCR method and cloned into the NheI and SpeI sites of the *cno*Δ*RA1-GFP* construct (forward primer, 5′-CGCATCTGAGCTAGCAAACTGTACACGGAACTACCAGAAACCTCGTTCACCCGATCG-3′; reverse primer, 5′-GCCTAGACTACTAGTCCGGGGCTGTGAGTCCGCCGGACGTC-3′). All rescue constructs were sequence verified. Each vector carrying the modified *cno* gene, pGE-attB-GMR, also carries a *w^+^* selectable marker next to the *cno* coding sequence, and both are flanked by attR and attL sites allowing site-specific integration into the attP site at the *cno*ΔΔ locus (Perez-Vale et al., 2021)*.* Injection of each mutant *cno*-*GFP* rescue construct was carried out by BestGene (Chino Hills, CA, USA) – DNA was injected into *PhiC31/int^DM. Vas^; cno*ΔΔ embryos (BDSC stock 94023). F1 offspring were screened for the presence of the *w^+^* marker and outcrossed to *w; TM6B, Tb/TM3, Sb* (BDSC stock 2537) to generate a balanced stock over TM3. We verified the integration of each mutant *cno-GFP* construct by both PCR amplification and sequencing and by immunoblotting. To remove potential other mutations from the mutant *cno-GFP* chromosomes we outcrossed each stock to a *y w* stock with a wild-type 3rd chromosome for multiple generations, selecting for the linked *w^+^* marker in each generation. This allowed us to homozygose *cno*Δ*RA2-GFP* and *cnoRA1-RA1-GFP.* All mutant *cno-GFP* stocks will be made available via the Bloomington Drosophila Stock Center upon publication.

### Embryo fixation and immunofluorescence

Embryo fixation and immunofluorescence were performed as described previously (McParland et al., 2024). Briefly, flies were placed into cups at 25°C with apple juice agar plates containing yeast paste. Using a paint brush, embryos were collected into 0.1% Triton X-100. Following a 5 min dechorionation with 50% bleach, embryos went through three 15 min washes in Triton salt solution (0.03% Triton X-100, 68 mM NaCl, EGTA), were fixed at 95°C for 10 s, and immediately cooled on ice for at least 30 min. Fixed embryos were devitellinized by vigorous shaking in a 1:1 solution of n-heptane and 95% methanol:5% EGTA. Devitellinized embryos were then transferred from the lower methanol layer into new tubes and washed three times with 95% methanol:5% EGTA. Prior to immunostaining, embryos went through three 15 min washes in phosphate-buffered saline (PBS) with 5% normal goat serum and 0.1% saponin (PBSS-NGS) and were blocked in PBSS-NGS for 1 h at room temperature. Antibodies were diluted in a PBS solution containing 1% bovine serum albumin and 0.1% saponin. Following primary antibody incubation either overnight at 4°C or 2–3 h at room temperature, embryos were washed three times for 15 min with PBSS-NGS and incubated in secondary antibodies for 2–3 h at room temperature. Antibodies and dilutions are in Table S2. Finally, embryos were washed three times with PBSS-NGS and mounted onto glass slides using a homemade Gelvatol solution (recipe from the University of Pittsburgh Center for Biological Imaging, https://cbi-pitt.webflow.io/protocols) and stored at 4°C.

### Image acquisition and analysis

Following fixation and immunostaining, embryos were imaged on an LSM 880 confocal laser-scanning microscope using a 40×/NA 1.3 Plan-Apochromat oil objective (Carl Zeiss, Jena Germany). ZEN 2009 software was used to acquire and process images and generate MIPs. Input levels, brightness and contrast were uniformly adjusted using Photoshop (Adobe, San Jose, CA, USA). Apical–basal positioning on MIPs was analyzed based on a method reported by Choi et al. (2013). *Z*-stack outputs (at 1.6× digital zoom) were cropped to 200×200 pixels in the region of interest in ZEN 2009 software. Dimensions of the regions of interest were converted from *yzx* to *xyz,* and MIPs were generated.

### Cno SAJ and TCJ/multicellular junction enrichment analysis

Images of *z*-stacks taken through the embryo with a digital zoom of 1.6× or 2× and a step size of 0.3 µm were used for SAJ and TCJ enrichment analysis. Cno TCJ intensity was analyzed in MIPs in stage 7 embryos. MIPs were generated in FIJI (National Institutes of Health, Bethesda, MD, USA) from the apical 1.2–2.4 µm region of *z*-stacks with a digital zoom of 1.6× or 2× and a step size of 0.3 µm. Lines were drawn (width of 5 pixels) in FIJI to measure mean Cno intensity of bicellular junctions (avoiding multicellular junctions), TCJs/multicellular junctions, and cytoplasm background signal at a 300% digital zoom. Cytoplasmic background was subtracted from all junctional signals to standardize pixel intensity for individual junctions. To calculate the Cno TCJ ratio, the mean standardized TCJ/multicellular junction intensity was divided by the mean standardized bicellular junction intensity (calculated from the average of all bicellular junctions in each TCJ). Ten multicellular junctions were analyzed per embryo yielding box and whisker plots showing 25th–75th percentile ratios in the box and whiskers showing 5th–95th percentiles. Graphs were produced and data statistical analyses were performed in GraphPad Prism version 10.0.0 (GraphPad Software, Boston, MA, USA) using Welch's unpaired *t*-test or Brown–Forsythe and Welch ANOVA test.

### Planar polarity quantification

Stage 7 to early stage 8 embryo apical 1.2–2.4 µm regions were measured to obtain planar polarity of Cno between AP and DV borders. Similar to multicellular junction analysis, lines were drawn (width of 5 pixels) at 300% zoom at bicellular borders splitting AP and DV axes and cytoplasm background signal to quantify pixel intensity using FIJI. Cytoplasmic background signal was subtracted from bicellular border intensity to generate standardized Cno intensity. Average AP border standardized Cno intensity was divided by average DV border standardized Cno intensity to generate an AP/DV Cno ratio to quantify planar polarity. Ten AP and ten DV borders were measured per embryo yielding box and whisker plots showing 25th–75th percentile ratios in the box and whiskers showing 5th–95th percentiles. Graphs were produced and data statistical analyses were performed in GraphPad Prism version 10.0.0 (GraphPad Software, Boston, MA, USA) using Welch's unpaired *t*-test or Brown–Forsythe and Welch ANOVA test.

### Cuticle preparation and analysis

We prepared embryonic cuticles as in Wieschaus and Nüsslein-Volhard (1986). Embryos were collected from apple juice agar plates with yeast paste and transferred to apple juice agar plates that incubated for at least 48 h at 25°C. Unhatched embryos were tallied and collected into 0.1% Triton X-100 where they were then dechorionated in 50% bleach for 5 min and washed three times with 0.1% Triton X-100. Unhatched embryos were transferred onto glass slides and mounted in a 1:1 solution of Hoyer's/lactic acid (Wieschaus and Nüsslein-Volhard, 1986) before being incubated at 60°C for 24–48 h and then stored at room temperature. Images were captured on an iPhone using a Nikon Labophot with a 10× Phase 2 lens and categorized based on morphological criteria.

### Immunoblotting

Table S2 contains the antibodies and dilutions used for these experiments. Expression levels of Cno, GFP and α-tubulin proteins were determined by immunoblotting embryonic lysates that were collected at 1–4 h and 12–15 h timepoints. Embryonic lysates were generated as in Manning et al. (2019). Briefly, embryos were collected into 0.1% Triton X-100, dechorionated for 5 min in 50% bleach and thrice washed with 0.1% Triton X-100. Subsequently, lysis buffer [1% NP-40, 0.5% Na deoxycholate, 0.1% SDS, 50 mM Tris-HCl pH 8.0, 300 mM NaCl, 1.0 mM DTT, Halt Protease and Phosphatase Inhibitor Cocktail (Thermo Fisher Scientific, #78442; 100×) and 1 mM EDTA] was added, and embryos were ground with a pestle for ∼20 s and placed on ice. After 10 min, embryos were again ground with a pestle for ∼20 s and subsequently centrifugated at 16,361 ***g*** for 15 min at 4°C. Protein concentration was assessed using Bio-Rad Protein Assay Dye and recording absorbance at 595 nm with a spectrophotometer. After resolving lysates using 7% SDS-PAGE, proteins were transferred onto nitrocellulose membranes. Prior to immunostaining, blocking was performed using 10% bovine serum albumin (BSA) diluted in Tris-buffered saline with 0.1% Tween-20 (TBST) for 1 h at room temperature. Primary and secondary antibodies were diluted in 5% BSA diluted in TBST. Primary antibody incubation was performed overnight at 4°C, and secondary antibody incubation was performed for 45 min at room temperature. The Odyssey CLx infrared system (LI-COR Biosciences) was used to image the membranes, and band densitometric analysis was performed using Empiria Studio Software (LI-COR Biosciences).

### Pupal eye dissection, immunofluorescence and analysis

Fly cultures were maintained at 25°C on nutrient-rich *Drosophila* medium, and pre-pupae were selected and maintained in humidified chambers until dissection at 40 h after puparium formation (DeAngelis and Johnson, 2019) Rabbit anti-Cno (1:500; Table S2) or chicken anti-GFP (1:8000, Abcam #13970) were used to detect Cno, with secondary antibodies obtained from Jackson ImmunoResearch (1:300; Table S2). Dissections were performed in triplicate, with 5–10 pupae of each genotype dissected each time. Images were gathered with a Leica DM5500 B fluorescence microscope and processed for publication using Adobe Photoshop, and patterning errors were scored in 8–14 retinas for each genotype as previously described (Johnson and Cagan, 2009). Student’s *t*-tests were used to determine statistical differences between the mis-patterning of different genotypes.

### Detailed author contributions

E.D.M. and N.J.G. carried out genetic and cell biological analyses of the various RA domain mutants, with help from C.C.J.; L.R.W. carried out biochemical analysis of the RA domains and their interactions with Rap1, with advice from K.C.S.; T.A.B. designed the RA domain mutants; N.J.G. carried out experiments to validate the allele and analyzed levels of protein expression; K.C.S. used AlphaFold data to examine predicted complexes of RA1 and RA2 with Rap1; A.B. and R.I.J. analyzed phenotypes in the developing eye; M.P. assisted with *Drosophila* genetics; and E.D.M., N.J.G., L.R.W., R.I.J., K.C.S. and M.P. wrote the manuscript with editing by the other authors.

## Supplementary Material

10.1242/joces.263546_sup1 Supplementary information
