# Supplementary material for: The dual Ras-association domains of Drosophila Canoe have differential roles in linking cell junctions to the cytoskeleton during morphogenesis
Source: J Cell Sci. 2024 Dec 11;137(23):jcs263546. doi: 10.1242/jcs.263546 (PMC11698047; doi:10.1242/jcs.263546)
Supplement: Supplementary information [file joces-137-263546-s1.pdf]

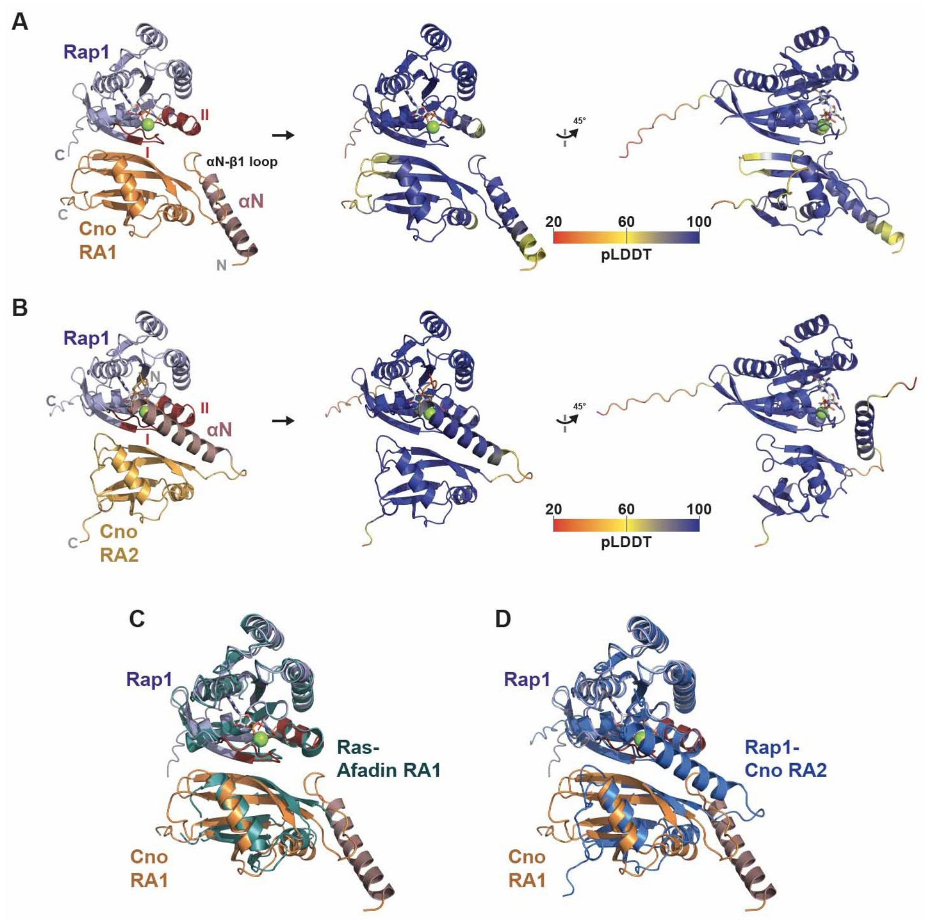

Fig. S1. AlphaFold2 structure predictions of Cno RA domain:Rap1 complexes have high confidence and align with the crystal structure of Afadin RA1:Ras. (A,B) AlphaFold2 structure models of the D.m. Cno RA1:Rap1 complex (A) and the D.m. Cno RA2:Rap1 complex (B) shown in cartoon format at left (as shown in Fig. 1 C,D), and colored according to prediction confidence represented by pLDDT value, right two panels, undergoing rotation as shown. (C) Structure alignment of the Drosophila Cno RA1:Rap1 AlphaFold2 structure prediction and the mouse Afadin RA1:human H-Ras-GMPPNP complex crystal structure (PDB ID 6AMB) (RMSD = 0.70 Å over 147 Cα atoms; (Smith et al., 2017). (D) Structure alignment of the Drosophila Cno RA1:Rap1 and RA2:Rap1 AlphaFold2 structure predictions (RMSD = 1.08 Å over 204 Cα atoms).

Journal of Cell Science • Supplementary information

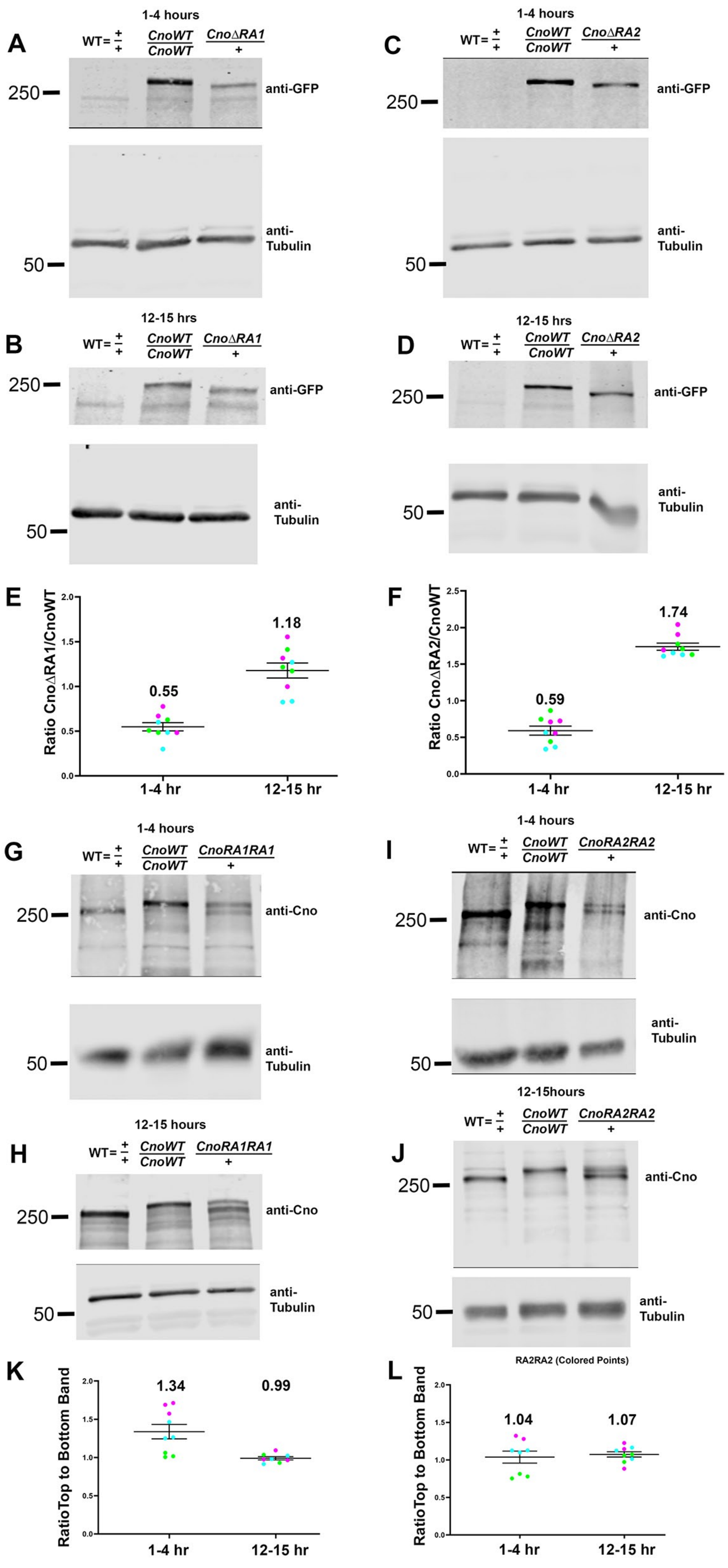

Journal of Cell Science • Supplementary information

Fig. S2. Our Cno mutant proteins accumulate at levels similar to wildtype. (A,B,C,D) Embryonic protein extracts of the indicated genotypes and timepoints, immunoblotted with antibodies to GFP, with α-tubulin as an internal loading control. The individual RA domains have a similar molecular weight to the C-terminal GFP fusion, thus causing GFP-tagged CnoΔRA1 and CnoΔRA2 proteins to run at an apparent molecular weight similar to wild-type Cno. Thus, for these genotypes we compared levels using the GFP-antibody, with cnoWT-GFP, which we previously found accumulates at levels similar to wildtype Cno, as our standard for quantifying mutant protein accumulation. Note that *cnoWT* (wildtype Canoe GFP tagged at the locus) is homozygous, while, because it is homozygous lethal, *cnoΔRA1* is heterozygous. The *cnoΔRA2* stock analyzed here is also heterozygous. We included this in our calculations. Thus, the apparent differences in the immunoblot are larger than the actual differences when both are homozygous. (E,F) Calculated levels of CnoΔRA1 and CnoΔRA2 relative to CnoWT. Band intensity values for CnoΔRA1 and CnoΔRA2 were multiplied by a factor of two to account for copy number difference compared to CnoWT. Technical replicates (dots) for each biological replicate (colors) are shown, with the wider band illustrating the mean value and the narrower bands representing the s.e.m. (G,H,I,J) Embryonic protein extracts of the indicated genotypes and timepoints, immunoblotted with antibodies to the C-terminus of Cno and α-tubulin as an internal loading control. *yellow white* (WT) and cnoWT-GFP (CnoWT) embryos were positive controls for Cno antibody. (K,L) Calculated levels of CnoRA1RA1 and CnoRA2RA2 relative to wild-type Cno. Due to sufficient separation between mutant and wild-type Cno bands, quantitation of CnoRA1RA1 and CnoRA2RA2 accumulation was achieved by evaluating the ratio of mutant Cno (top band) to wild-type Cno (bottom band). Technical replicates (dots) for three biological replicates (colors) are shown, with the wider band illustrating the mean value and the narrower bands representing the s.e.m.

Journal of Cell Science • Supplementary information

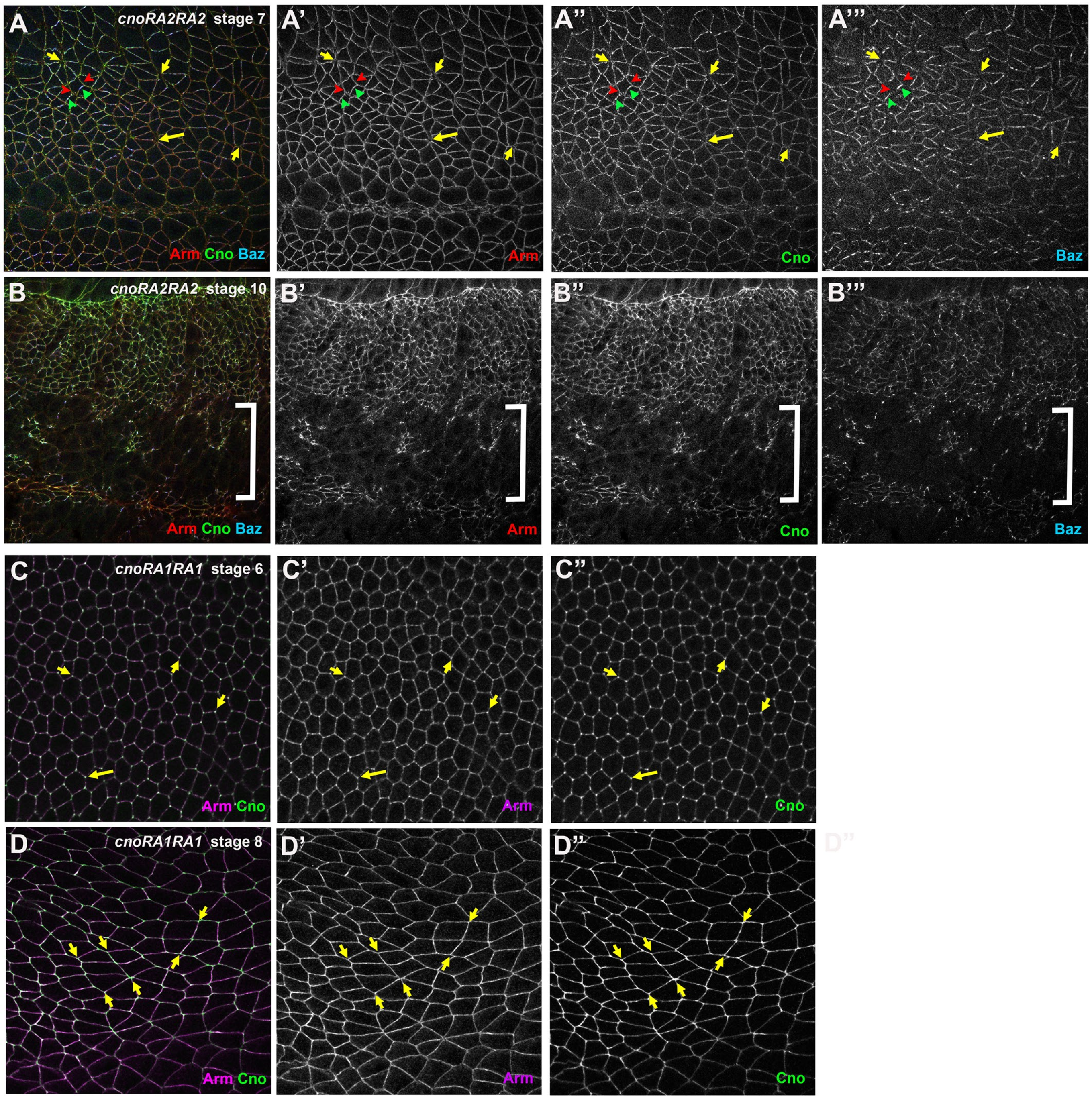

Fig. S3. *cnoRA2RA2* mutants have severe defects in junction stabilization and junctional protein localization, while *cnoRA1RA1* mutants are apparently wildtype in localization and function. Embryos, anterior left and dorsal up, stage, genotype and antigens indicated. (A,B) *cnoRA2RA2* mutants. (A) Mutants have junctional gaps at rosette centers and aligned AP borders (yellow arrows). Baz planar polarity is enhanced, with loss on AP borders (red arrowheads) but retention on DV borders (green arrowheads). (B) In *cnoRA2RA2* mutants the ventral epidermis loses epithelial integrity (bracket) –Arm and Cno are reduced in rounded up cells and in other cells junctions are fragmented. (C,D) *cnoRA1RA1* mutants. (C) At stage 6 CnoRA1RA1 protein is correctly enriched at tricellular junctions (yellow arrows). (D) At stage 8 CnoRA1RA1 protein is correctly enriched at aligned AP borders (yellow arrows).

Journal of Cell Science • Supplementary information

Table S1. Patterning defects per ommatidium (N=76 to 110 ommatidia per genotype)

Genotype	Cone cell defects		Primary cell defects		Ommatidial misorientation		Bristle defects		Tertiary cell defects		Number of lattice cells		Mis-patterning score			Mis-patterning score comparison (p-value, * = significant)	
	Mean	Std Dev	Mean	Std Dev	Mean	Std Dev	Mean	Std Dev	Mean	Std Dev	Mean	Std Dev	Mean	Std Dev	Std Error		
w^{1118}; cno-wt::GFP	**0.00**	0.00	**0.00**	0.00	**0.00**	0.00	**0.20**	0.40	**0.25**	0.47	**11.95**	0.51	**0.71**	1.20	0.14		
w^{1118}; cno^{R2} / +	**0.04**	0.21	**0.00**	0.00	**0.00**	0.00	**0.03**	0.18	**0.08**	0.27	**12.27**	0.54	**0.45**	0.78	0.08	0.1063	compared to *cno-wt::GFP*
w^{1118}; cnoRA1RA1::GFP	**1.06**	1.12	**0.12**	0.40	**0.01**	0.11	**0.27**	0.45	**0.35**	0.60	**11.75**	1.03	**2.54**	2.44	0.27	**1.9209×10^{-8} ***	compared to *cno-wt::GFP*
w^{1118}; cno-ΔRA2::GFP	**0.25**	0.54	**0.10**	0.56	**0.00**	0.00	**0.13**	0.34	**0.22**	0.42	**11.69**	0.76	**1.14**	1.43	0.14	0.0313	compared to *cno-wt::GFP*
w^{1118}; cno-ΔRA2::GFP / cno^{R2}	**0.82**	0.75	**0.25**	0.81	**0.08**	0.27	**0.34**	0.53	**0.41**	0.59	**11.45**	0.90	**2.61**	2.62	0.25	**0.8291×10^{-8} ***<br>**0.0265×10^{-8} ***	compared to *w^{1118} ; cno-ΔRA2::GFP*<br>compared to cno-wt::GFP

Table S2. Antibodies Used

Primary Antibodies	Host Species	Dilution	Source
Anti-Armadillo	Mouse IgG_{2a}	1:100 (IF)	Developmental Studies Hybridoma Bank (N2 7A1)
Anti-α-tubulin	Mouse IgG_1	1:5000 (WB)	Sigma-Aldrich (T6199)
Anti-Bazooka	Rabbit IgG	1:2000 (IF)	Choi et al., 2013
Anti-Canoe	Rabbit IgG	1:1000 (IF)<br>1:2000 (WB)	Sawyer et al., 2009
Anti-GFP (JL-8)	Mouse IgG_{2a}	1:1000 (WB)	Clontech Laboratories (632381)
Anti-GFP	Chicken IgY	1:1000 (IF)	Rockland Immunochemicals, Inc. (600-901-215)
**Secondary Antibodies**	**Host Species**	**Dilution**	**Source**
Anti-Mouse IgG_{2a} Alexa Fluor 647	Goat IgG	1:1000 (IF)	ThermoFisher Scientific (A-21241)
Anti-Rabbit IgG Alexa Fluor 568	Goat IgG	1:1000 (IF)	ThermoFisher Scientific (A-11011)
Anti-Chicken IgY Alexa Fluor 488	Goat IgG	1:1000 (IF)	ThermoFisher Scientific (A-11039)
Anti-Rabbit IgG Alexa Fluor 488	Donkey IgG	1:300 (IF)	Jackson ImmunoResearch (711-545-152)
Anti-Rabbit IgG IRDye 680RD	Goat IgG	1:10000 (WB)	LI-COR Biosciences (926-68071)
Anti-Mouse IgG IRDye 800CW	Donkey IgG	1:10000 (WB)	LI-COR Biosciences (926-32212)

Journal of Cell Science • Supplementary information
